# Transition-metal-catalyzed domino reactions of strained bicyclic alkenes

**DOI:** 10.3762/bjoc.19.38

**Published:** 2023-04-24

**Authors:** Austin Pounder, Eric Neufeld, Peter Myler, William Tam

**Affiliations:** 1 Guelph-Waterloo Centre for Graduate Work in Chemistry and Biochemistry, Department of Chemistry, University of Guelph, Guelph, Ontario, N1G 2W1, Canada

**Keywords:** bicyclic alkenes, cascade, catalysis, domino, transition-metal-catalyzed

## Abstract

This review presents a comprehensive overview of transition-metal-catalyzed domino reactions of strained bicyclic alkenes, including both homo- and heterobicyclic alkenes. These compounds are important synthons in organic synthesis, providing an important platform for the construction of biologically/medicinally significant compounds which bear multiple stereocenters. The review has been divided according to the metal used in the reaction. An overview of the substrate scope, reaction conditions, and their potential applications in organic synthesis is discussed. A comprehensive outlook on the reactivity paradigms of homo- and heterobicyclic alkenes is discussed and should shed light on future directions for further development in this field.

## Introduction

A well-orchestrated sequence of events – cascade, also known as domino, tandem, and sequential reactions, constitutes a fascinating branch of organic chemistry dedicated to the synthesis of highly functionalized products through sequential transformations in a single reaction. Classically, a domino reaction has been defined by Tietze as a reaction involving two or more bond-forming transformations that take place under the same reaction conditions, without adding additional reagents and catalysts, and in which the subsequent reactions result as a consequence of the functionality formed in previous steps [1].

**Figure 1:**
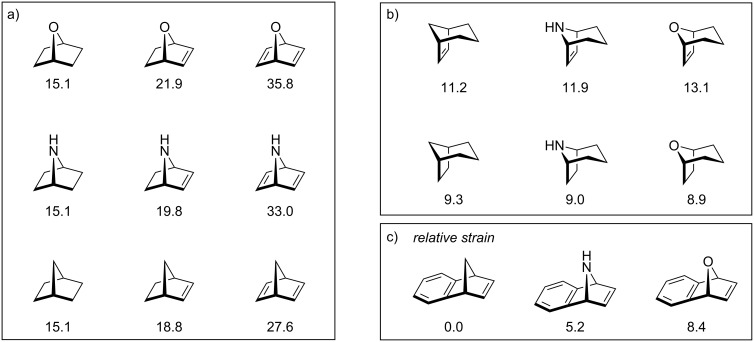
Ring-strain energies of homobicyclic and heterobicyclic alkenes in kcal mol^−1^. a) [2.2.1]-Bicyclic systems. b) [2.3.1]-Bicyclic systems. c) Benzo-fused [2.2.1]-bicyclic systems; ring-strain energy relative to benzonorbornadiene.

Bicyclic alkenes, a family of strained ring systems, have seen widespread applications in organic synthesis in the last 20 years [2–6]. Broadly speaking, bicyclic alkenes can be classified into two groups: homobicyclic and heterobicyclic alkenes. Homobicyclic alkenes are hydrocarbons, like norbornadiene, while heterobicyclic alkenes contain at least one heteroatom in the bicyclic framework. Typically, reactions involving these strained bicyclic alkenes are thermodynamically driven forward with the release of ring-strain energy (Figure 1) [7–8]. Intuitively, increasing the number of olefin moieties in the bicyclic system from zero, one, and two, increases the ring-strain energy. Moreover, the introduction of a bridging heteroatom increases the ring-strain energy of the system, conceptualized by the decrease in bond distances. Typically, there are two modes for ring-strain release. First, functionalization of the double bond mildly alleviates the ring strain by relieving nonoptimal bond angles enforced by the rigid bicyclic framework. Secondly, through ring opening of the bicyclic framework; the C–X bond of a heterobicyclic alkene is much weaker than the corresponding C–C bond of a homobicyclic alkene, which allows the C–X bond to be readily cleaved over the course of a reaction.

**Figure 2:**
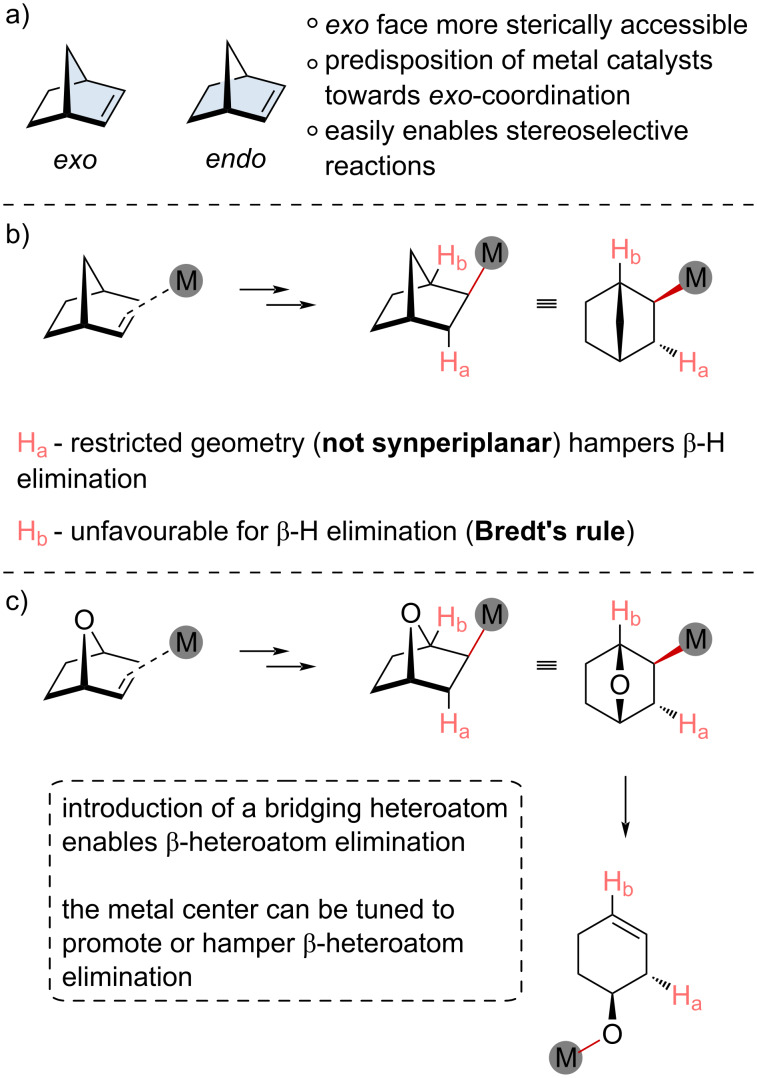
a) *Exo* and *endo* face descriptions of bicyclic alkenes. b) Reactivity comparisons for different β-atom elimination steps for an *exo-*metallated intermediate carbobicyclic system. c) Reactivity comparisons for different β-atom elimination steps for an *exo-*metallated intermediate oxabicyclic system.

The stereochemically well-defined and rigid nature of these bicyclic alkenes creates two diastereotopic faces, namely the *endo* and *exo* face (Figure 2). The *exo* face is sterically less congested than the *endo* face; therefore, the *exo* face will typically interact with metal catalysts through side-on coordination of the olefin, and in the case of heterobicyclic alkenes, the heteroatom. This preferential *exo* coordination is not always the case, as norbornadiene derivatives are known to preferentially form chelated *endo* complexes which can change the stereochemical outcome of the reaction. Nevertheless, the predisposition of metal catalysts towards coordination on the *exo* face biases the reaction outcome towards *exo*-selective functionalization.

Upon *exo* coordination of a metal catalyst with the π system and subsequent migratory insertion, the resulting alkyl metal intermediate is quite limited in how it can propagate. In the case of a carbobicyclic system (Figure 2b), the rigidity of the bicyclic framework restricts β-H elimination. The inability to rotate to achieve an optimal synperiplanar geometry restricts efficient elimination (Figure 2b, H_a_). Bridgehead protons are in a more favorable geometry for β-H elimination (Figure 2b, H_b_); however, their elimination would generate a highly unstable alkene at the bridgehead, violating Bredt’s rule [9]. For these reasons, carbobicyclic alkenes have been exploited as propagation mediators, as seen in Catellani-type reactions [10–12]. In this review, we will focus on the functionalization of the bicyclic framework itself rather than its use as a transient mediator for domino reactions [13–17]; however, we point the reader to several excellent reviews. The “trapped” alkyl metal intermediate can undergo subsequent migratory insertion steps with other π systems or can be intercepted by an electrophile.

The introduction of a bridging heteroatom into the bicyclic scaffold can dramatically alter the reactivity (Figure 2c). Besides the apparent increase in the ring strain (vide supra), their potential propagation steps are more complex. After an *exo* coordination of a metal catalyst with the π system and migratory insertion, the resulting heterobicyclic alkyl metal intermediate is not as kinetically stable as its carbocyclic counterpart. While β-H elimination is still limited, these heterobicyclic alkenes will often undergo β-heteroatom elimination to generate ring-opened intermediates (Figure 2c). Fortunately, the metal center can be tuned to promote or hamper the β-H elimination, providing two routes for reaction propagation: ring opening and interception of the ring-opened intermediate or functionalization of the alkyl metal intermediate.

Throughout the past decade, research efforts have demonstrated a broad range of strained bicyclic alkenes can be exploited in domino reactions to selectively generate highly functionalized ring systems. Over the years, several different metal catalysts have been used, each allowing for a breadth of unique coupling partners to either propagate the reaction or to terminate the process.

This review presents a comprehensive examination of domino reactions involving strained bicyclic alkenes. Rather than being exhaustive in the range of potential difunctionalization processes covered, the review will be limited to domino reactions which include at least two distinct reactions. The review is divided on the basis of the transition-metal catalyst used in the reaction and will not cover metal-free methods. The literature is covered up to and including January 2023. For reasons of clarity, newly formed bonds are sketched in red, with newly formed cyclic structures being highlighted.

## Review

### Earth-abundant metals

Among the transition metal used in organic synthesis, the late transition metals like rhodium, palladium, and iridium have taken center stage when it comes to methodology development. Although these late-stage transition metals have contributed immensely to synthetic organic and organometallic chemistry, increasing societal awareness in terms of sustainable developments and resource management has prompted chemists to explore the use of environmentally benign, inexpensive, and earth-abundant metals [18–27]. In this section, we summarize recent progress in Ni, Fe, Cu, and Co-catalyzed domino reactions of strained bicyclic alkenes.

#### Nickel-catalyzed reactions

Without close inspection, nickel might seem like the peculiar younger sibling of palladium within the field of transition-metal catalysis. Nickel lies directly above palladium in the periodic table, as such, it readily performs many of the same elementary reactions. Because of their reactive commonalties, nickel is often seen as the budget-friendly replacement; however, this misconception will clearly be refuted in this section, showcasing several diverse nickel-catalyzed domino reactions.

**Scheme 1:**
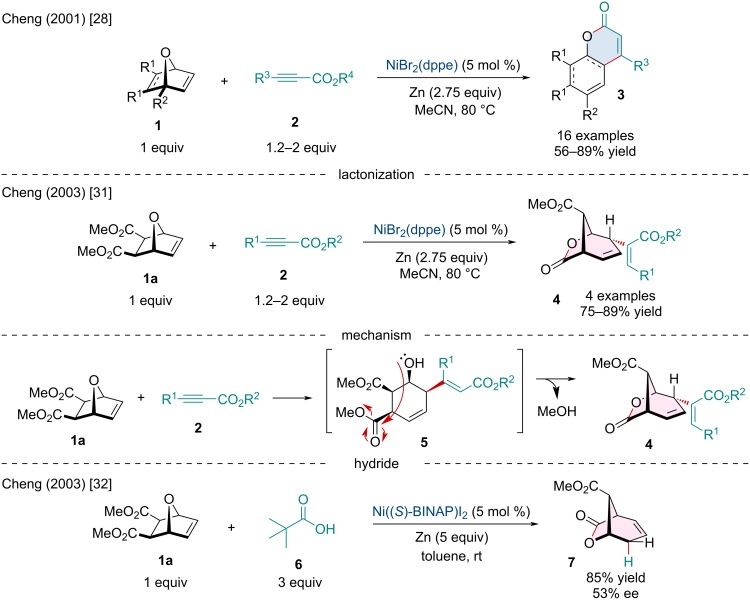
Ni-catalyzed ring-opening/cyclization cascade of heterobicyclic alkenes **1** with alkyl propiolates **2** to generate coumarin **3** and bicyclic γ-lactone **4** derivatives.

In 2001, Rayabarapu and co-workers investigated the Ni-catalyzed ring-opening/cyclization cascade of heterobicyclic alkenes **1** with alkyl propiolates **2** for the synthesis of coumarin derivatives **3** (Scheme 1) [28]. The reaction initiates with the in situ reduction of Ni(II) to Ni(0) followed by the side-on coordination of the alkene and alkyne substrates to the metal center with subsequent oxidative cyclometallation to form a nickel metallacycle, similar to several reported Ni-catalyzed [2 + 2] cycloadditions [29–30]. Rather than undergoing reductive elimination to afford to [2 + 2] adduct, β-oxygen elimination followed by *E/Z* isomerization and intramolecular lactonization generates the annulated coumarin scaffold. In 2003, the Cheng lab extended on this Ni-catalyzed ring-opening strategy [31]. It was noted the addition of 1.5 equivalents of water interrupted the cyclization step and led entirely to reductively coupled alkenylated ring-opened products. Interestingly, when this methodology was applied to the ester-bearing oxabicyclic **1a**, the anticipated reductive coupling product was not detected; instead, bicyclic γ-lactone **4** was solely observed (Scheme 1). This unprecedented lactone is presumed to be generated through the expected reductive coupling to generate the ring-opened intermediate **5** which undergoes subsequent intramolecular lactonization with the distal ester group. In the same year, Cheng and co-workers observed the identical reactivity when exploring the Pd- and Ni-catalyzed asymmetric reductive ring opening of heterobicyclic alkenes, ultimately generating the bicyclic product **7** (Scheme 1) [32].

**Scheme 2:**
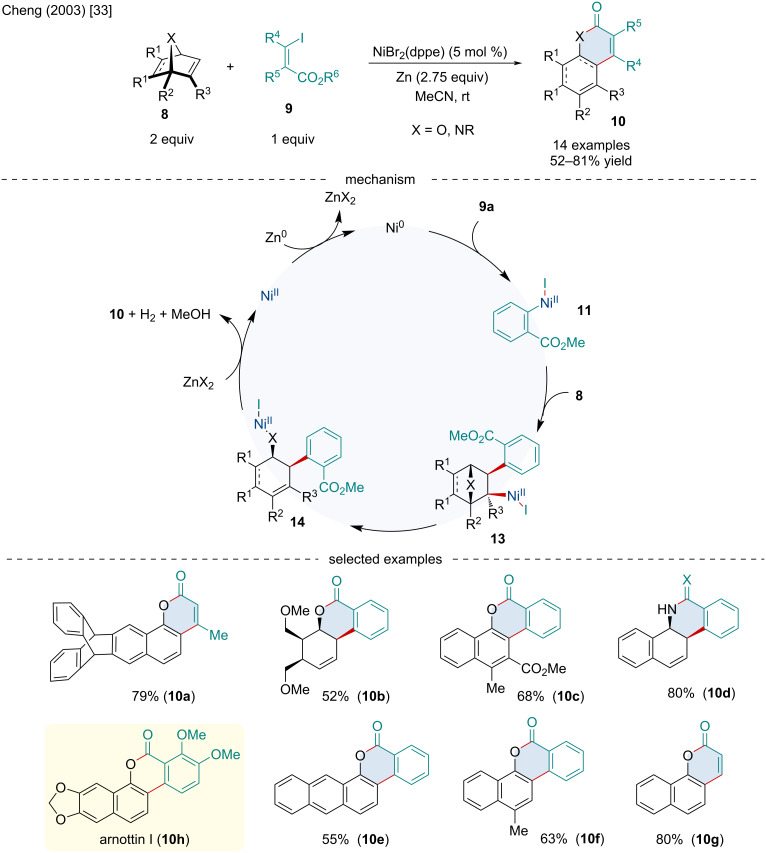
Ni-catalyzed ring-opening/cyclization cascade of heterobicyclic alkenes **8** with β-iodo-(Z)-propenoates and *o*-iodobenzoates **9**.

In 2003, the Cheng laboratory continued studying Ni-catalyzed routes towards coumarin cores through the Ni-catalyzed ring-opening/cyclization cascade of heterobicyclic alkenes **1** with β-iodo-(*Z*)-propenoates and *o*-iodobenzoates **9** (Scheme 2) [33]. The authors noted the ring-opening/cyclization cascade proceeded smoothly for a variety of heterobicyclic alkenes including both oxa- and azabenzonorbornadienes as well as oxanorbornenes; however, the latter two substrates did not undergo dehydrogenation, generating *cis*-selective annulated coumarins (**10b** and **10d**). In 2006, the same group applied this methodology for the total synthesis of arnottin I (**10h**), a coumarin-type natural product isolated from the bark of the *Xanthoxylum arnottianum Maxim* which possesses some antibiotic properties [34]. Mechanistically, the authors proposed the reaction begins with the in situ reduction of Ni(II) to Ni(0) by zinc to generate Ni(0) which undergoes oxidative addition with the organo iodide to yield Ni(II) intermediate **11**. Coordination of **11** to the bicyclic alkene followed by migratory insertion affords intermediate **12** which undergoes β-oxygen elimination to form **13**. Rearrangement of **13** via β-hydride elimination and enolization generates a 1-naphthol species which undergoes intramolecular cyclization with the ester to form the final product **10**. The selectivity for the non-dehydrogenated coumarins **10d** is not understood, but **10b** likely does not undergo dehydrogenation because there is no formation of aromaticity to drive the reaction forward. When the bicyclic alkene is substituted unsymmetrically at the bridgehead position, the reaction is entirely regioselective for the formation of a 1,2,4-trisubstituted pattern. The observed regioselectivity arises from the preferential migratory insertion of the aryl group distal to the bridgehead substituent.

**Scheme 3:**
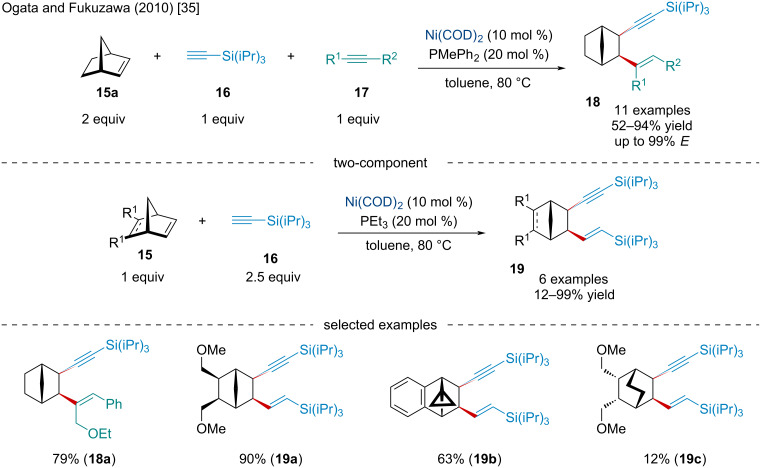
Ni-catalyzed two- and three-component difunctionalizations of norbornene derivatives **15** with alkynes **16** and **17**.

In 2010, Ogata and Fukuzawa explored the Ni-catalyzed two- and three-component difunctionalization of norbornene derivatives **15** with alkynes (Scheme 3) [35]. It was noted the reaction is amenable to both electron-donating groups (EDGs) and electron-withdrawing groups (EWGs); however, yields were diminished with increasing electron deficiency. Moreover, the use of the bulkier *tert*-butyldimethylsilyl-protecting group resulted in the corresponding 1,5-enyne only being produced in a 33% yield. Several different norbornene derivatives were explored and gave the anticipated *exo,exo-*difunctionalized product in good yield. In contrast, when using an ethylene-bridged bicycloalkene to generate the product **19c**, the latter was obtained in a greatly reduced yield, perhaps due to less ring strain providing a thermodynamic driving force.

**Scheme 4:**
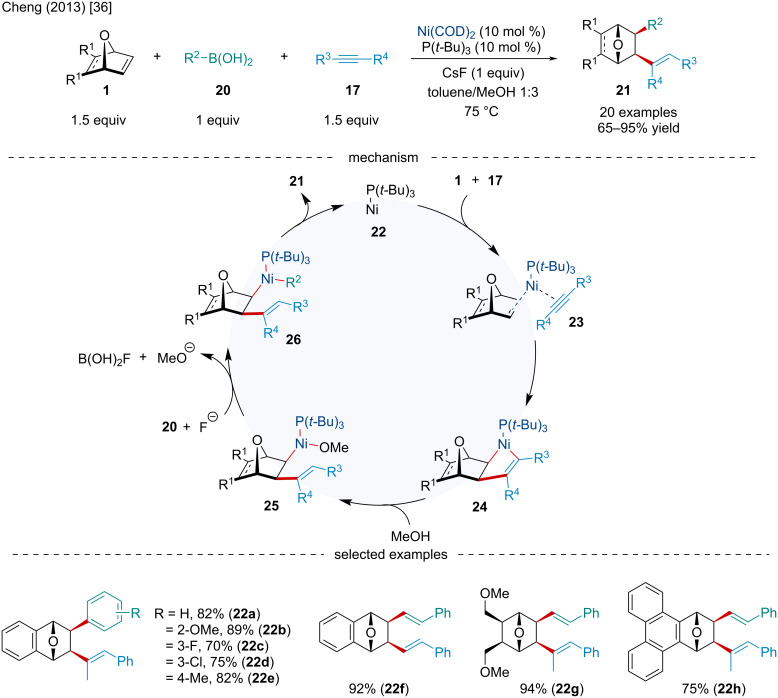
Ni-catalyzed intermolecular three-component difunctionalization of oxabicyclic alkenes **1** with alkynes **17** and organoboronic acids **20**.

In 2013, Mannathan et al. discussed a Ni-catalyzed intermolecular three-component difunctionalization of oxabicyclic alkenes **1** with organoboronic acids **20** and alkynes **17** (Scheme 4) [36]. While broadly successful, when electron-deficient arylboronic acids were used, slightly diminished yields were observed. Moreover, when 3-hexyne was used, the reaction failed to afford any product. The reaction likely begins similarly to Cheng’s 2003 report (Scheme 1) [31] where the coordination of the alkyne **17** and alkene **1** to the Ni(0) center, followed by oxidative cyclometallation, yields the following nickelocycle **24**. Unlike Cheng’s 2003 report, which proposes subsequent β-oxygen elimination (Scheme 1) [31], alkoholysis by MeOH affords an alkyl(methoxy)nickel intermediate **25**. Transmetalation of **25** with the organoboronic acid gives intermediate **26**, which upon reductive elimination affords the difunctionalized product **21** and regenerates the Ni(0) catalyst.

**Scheme 5:**
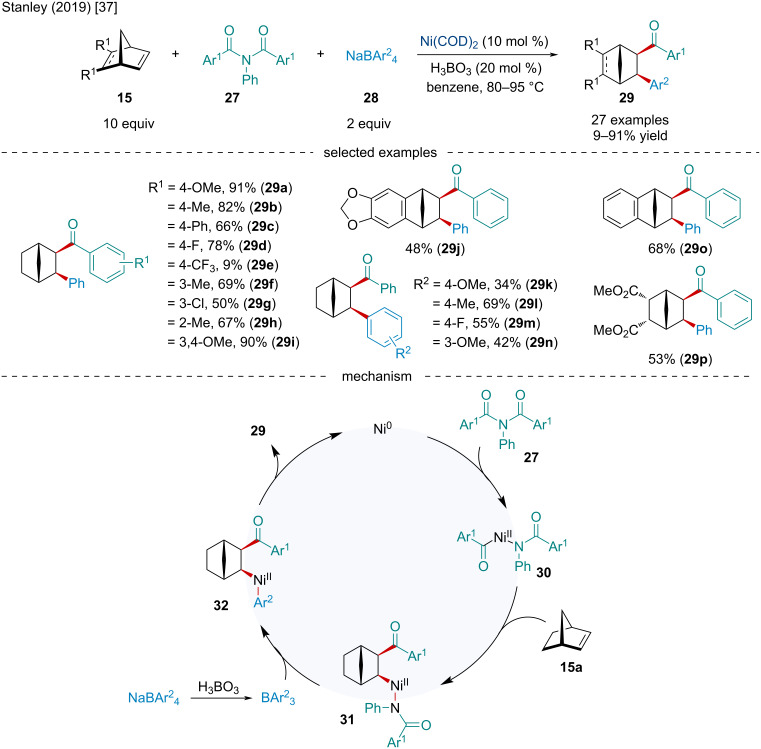
Ni-catalyzed intermolecular three-component carboacylation of norbornene derivatives **15**.

In 2019, the Stanley laboratory explored the Ni-catalyzed intermolecular three-component carboacylation of norbornene derivatives **15** using imides **27** and tetraarylborates **28** (Scheme 5) [37]. The method utilizes C–N bond activation to trigger the reaction. The authors demonstrated a broad reaction scope. Electron-deficient amides were shown to perform worse than their electron-rich counterparts with the *p*-trifluoromethyl substituent forming the ketone product in <10% yield. While substitution of the norbornene was tolerated, both EWGs and EDGs hindered the reaction. Upon several mechanistic studies, the authors proposed the catalytic cycle begins with the oxidative addition of the active Ni(0) catalyst to imide **27** to afford the acyl–Ni(II)–amido intermediate **30**. Side-on coordination followed by migratory insertion of the bicyclic alkene selectively generates the *exo*-alkyl–Ni(II)–amido complex **31**. Transmetalation with triarylborane affords **32** which undergoes reductive elimination to form the carboacylated product **29** as well as regenerates the Ni(0) catalyst. In 2022, the Tobisu group explored a two-component carboacylation of norbornene derivatives. Exploiting a Ni/NHC system, the authors were able to develop an entirely atom-economic carboacylation process utilizing *N-*indoyl arylamides [38].

**Scheme 6:**
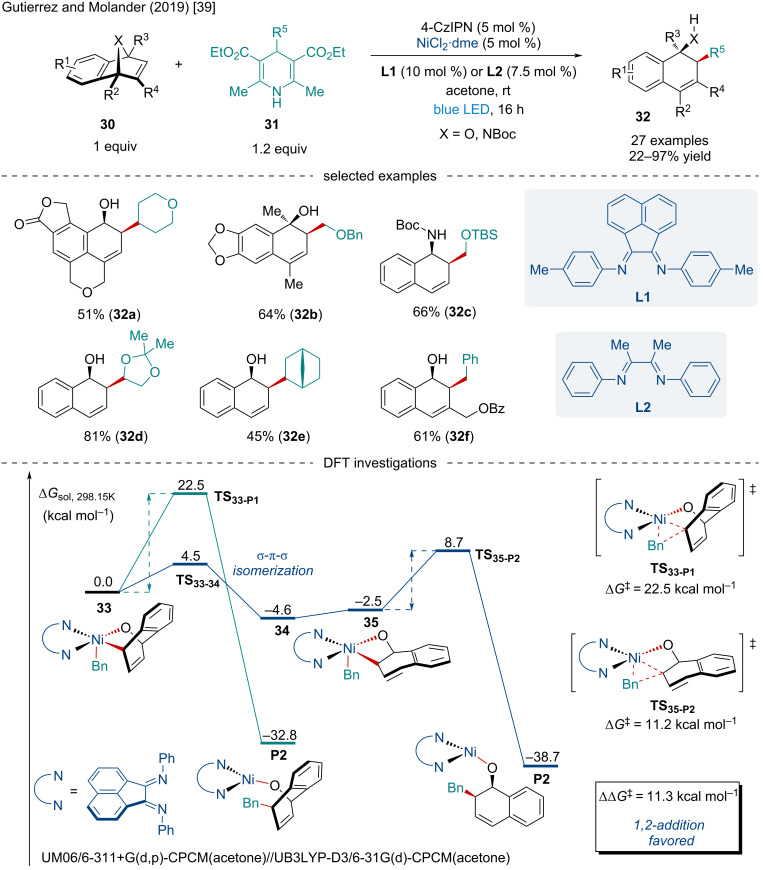
Photoredox/Ni dual-catalyzed coupling of 4-alkyl-1,4-dihydropyridines **31** with heterobicyclic alkenes **30**.

**Scheme 7:**
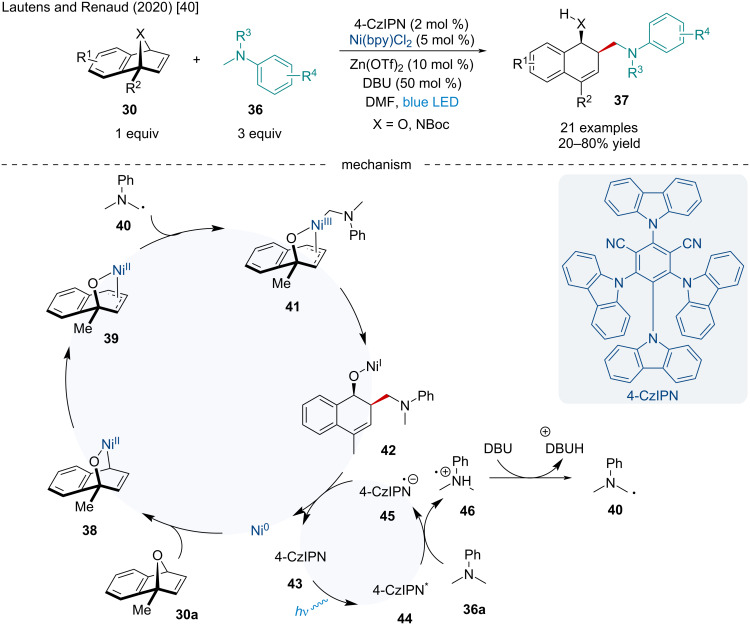
Photoredox/Ni dual-catalyzed coupling of α-amino radicals with heterobicyclic alkenes **30**.

In 2019, Gutierrez and Molander reported the coupling 4-alkyl-1,4-dihydropyridines **31** with heterobicyclic alkenes **30** under photoredox/Ni dual catalysis (Scheme 6) [39]. In contrast to other photoredox-mediated transformations, the authors utilized the inexpensive organic photosensitizer 4-CzIPN (Scheme 6 and Scheme 7) instead of the more commonly, and expensive, metal-based photocatalysts. While broadly successful, tertiary radicals failed to deliver any desired product. Of note, the reaction was amenable to a broad scope of derivatized heterobicyclic alkenes with mono- and disubstituted bridgeheads having little effect on the reaction (**32b**) with reactions involving unsymmetrically substituted bicyclic alkenes demonstrating complete regioselectivity for either 1,2,3- or 1,2,4-trisubstitued products (**32a**, **32f**). DFT calculations were used to explain the *syn*-1,2-substitution experimentally observed rather than the possible *syn*-1,4-substituted product. It was found the reductive elimination transition state leading to the 1,4-disubstituted product **TS_33-P1_** would require an increase in distortion energy compared to **TS_35-P2_** which contributes to an overall greater kinetic barrier.

The following year, Lautens and Renaud expanded the scope of the photoredox/Ni dual-catalyzed coupling of alkyl nucleophiles **36** with heterobicyclic alkenes **30** to include α-amino radicals (Scheme 7) [40]. The authors noted the electron-rich oxabenzonorbornadiene derivatives provided the corresponding ring-opened adducts in good yields (63–68% yield) while those bearing EWG led to poor product formation. Unlike Gutierrez and Molander’s work (Scheme 6) [39], it was found mono- and disubstituted bridgeheads affected the efficacy of the reaction with the demethylated bridgehead oxabenzonorbornadiene only delivering the product in a 20% yield. Although yields were slightly diminished, unsymmetrical bridgehead-monosubstituted oxabenzonorbornadiene led solely to the 1,2,4-trisubstituted regioisomer (Scheme 7), similar to that observed by Gutierrez and Molander [39]. Selected substituents on the aniline motif were found to hamper reactivity with a few examples failing to provide the desired product when 4-CzIPN was used as the photocatalyst; however, the products were isolated when [Ir(dF(CF_3_)ppy)_2_(bpy)]PF_6_ was used. Based on experimental observations and control reactions, the authors proposed the reaction begins with the photoexcitation of the photosensitizer **43** to form **44** which can oxidize aniline **36a** to give radical cation **46** (Scheme 7). Deprotonation by DBU produces the radical **40**. The radical anion photosensitizer **45** can reduce Ni(I) to Ni(0), closing the first catalytic cycle. The Ni(0) complex can undergo oxidative addition into the C–O bond of the oxabicyclic alkene **30a** to afford the σ-allyl intermediate **38** which can isomerize to the more stable π-allyl intermediate **39**. Addition of the α-amino radical to the Ni(II) center generates the Ni(III) complex **41**. Reductive elimination, followed by protodemetalation, leads to the final ring-opened adduct **37**.

#### Copper-catalyzed reactions

**Scheme 8:**
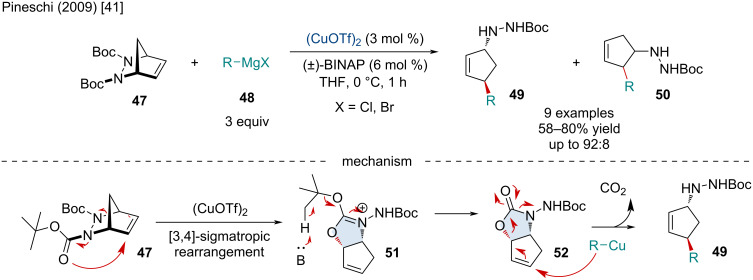
Cu-catalyzed rearrangement/allylic alkylation of 2,3-diazabicyclo[2.2.1]heptenes **47** with Grignard reagents **48**.

In 2009, Pineschi and co-workers explored the Cu-catalyzed rearrangement/allylic alkylation of 2,3-diazabicyclo[2.2.1]heptenes **47** with Grignard reagents **48** (Scheme 8) [41]. The reaction is thought to proceed via the Lewis acid-catalyzed [3,4]-sigmatropic rearrangement of the diazabicycle **47** to form the allylic carbazate intermediate **51**. Nucleophilic attack of an organomagnesium, or organocuprate, in an *anti* S_N_2’ fashion on **52** furnish the final ring-opened product **49**. The authors note the use of a carbamate protecting group was crucial for the success of the reaction, hypothesizing it inhibited the classical [3,3]-sigmatropic Lewis acid-catalyzed rearrangement often observed. Both alkyl and aryl Grignard reagents were amenable to the reaction; however, heteroaryl Grignard reagents resulted in poor conversion.

The Cu-catalyzed borylative difunctionalization of π-systems is a power tool for the facile synthesis of complex boronate-containing compounds [42]. Generally, these reactions proceed through the generation of a Cu–boryl species via σ-bond metathesis, followed by migratory insertion with a π-system. The subsequent alkyl–Cu intermediate is intercepted by an electrophile to generate the difunctionalized system. This methodology has been applied several times to strained bicyclic alkenes with a variety of electrophiles.

**Scheme 9:**
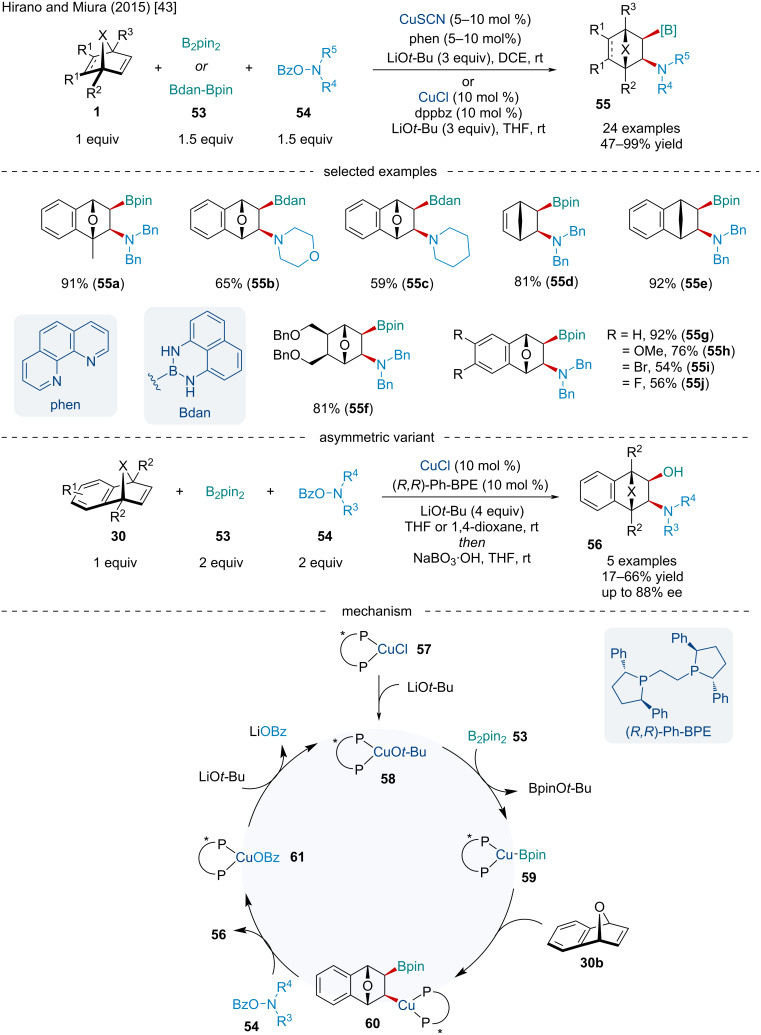
Cu-catalyzed aminoboration of bicyclic alkenes **1** with bis(pinacolato)diboron (B_2_pin_2_) (**53**) and *O*-benzoylhydroxylamine derivatives **54**.

In 2015, Hirano and Miura developed a Cu-catalyzed aminoboration of bicyclic alkenes **1** with bis(pinacolato)diboron (B_2_pin_2_) (**53**) and *O*-benzoylhydroxylamine derivatives **54** (Scheme 9) [43]. While the scope of bicyclic alkenes was quite extensive with aza-, carbo-, and oxabicyclic alkenes being amenable to the reaction, electron-deficient substrates resulted in lowered yields. Of note, the reaction is highly regioselective with the unsymmetrically methyl-substituted bicyclic alkene producing a single regioisomer **55a**. The authors noted the aminoborylated products bearing a BPin moiety were not always stable upon isolation, so they were either converted into the more stable Bdan (dan = 1,8-diaminonaphthalenyl) or Bpin-Bdan was used directly which showed comparable yields. The authors also reported preliminary results for an asymmetric variant of the reaction using (*R,R*)-Ph-BPE as a chiral ligand. Although the use of the chiral phosphine ligand resulted in slightly diminished yields, the authors were able to achieve ees up to 88%. The authors proposed the reaction begins with the generation of the *tert*-butoxide Cu salt which undergoes σ-bond metathesis with B_2_Pin_2_ generating the Cu–boryl species **59** (Scheme 9). Side-on coordination on the *exo* face of the bicyclic alkene followed by migratory insertion generates the alkyl–Cu species **60** which after electrophilic amination with the *O*-benzoylhydroxylamine **54** liberates the final aminoborylated product **55** and a benzoyl–Cu complex **61**. To close the catalytic cycle a transmetalation of **61** with LiO*t-*Bu regenerates the active catalyst.

**Scheme 10:**
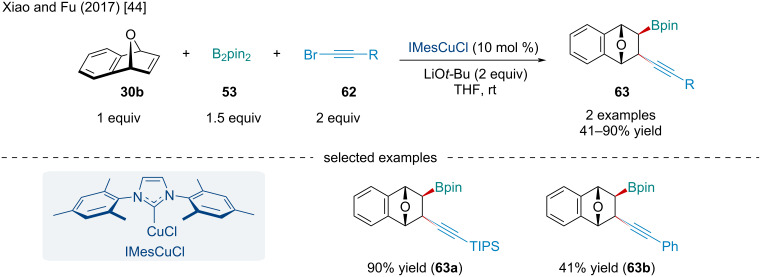
Cu-catalyzed borylalkynylation of oxabenzonorbornadiene (**30b**) with B_2_pin_2_ (**53**) and bromoalkynes **62**.

In 2017, Xiao and Fu studied the Cu-catalyzed borylalkynylation of oxabenzonorbornadiene (**30b**) with B_2_pin_2_ (**53**) and bromoalkynes **62** (Scheme 10) [44]. The scope of the reaction was limited to only two examples of bromoalkynes reacting with oxabenzonorbornadiene (**30b**). Notably, the yield of the reaction dramatically diminished when the terminal triisopropylsilyl (TIPS) group in **63a** was swapped for a Ph (**63b**). Mechanistically, the reaction operates in a similar manner reported by Hirano and Miura (Scheme 9) [43]; however, the alkyl–Cu species **60** is intercepted by the bromoalkyne rather than an *O*-benzoylhydroxylamine.

**Scheme 11:**
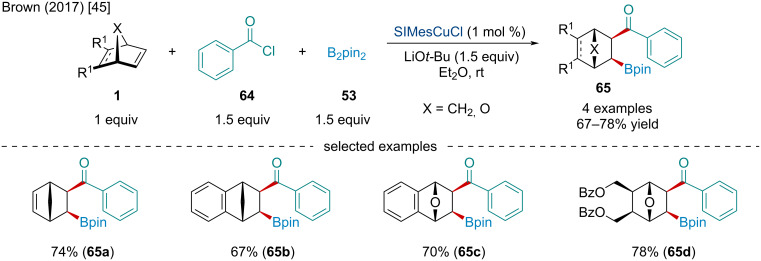
Cu-catalyzed borylacylation of bicyclic alkenes **1**.

In the same year, the Brown laboratory investigated the Cu-catalyzed borylacylation of bicyclic alkenes **1** (Scheme 11) [45]. Like the previous borylative difunctionalization reactions, it was found the reaction generated a single *exo,exo* diastereomer. A brief investigation into an enantioselective variant of the borylacylation was investigated; however, the methodology was not applied to bicyclic alkenes.

**Scheme 12:**
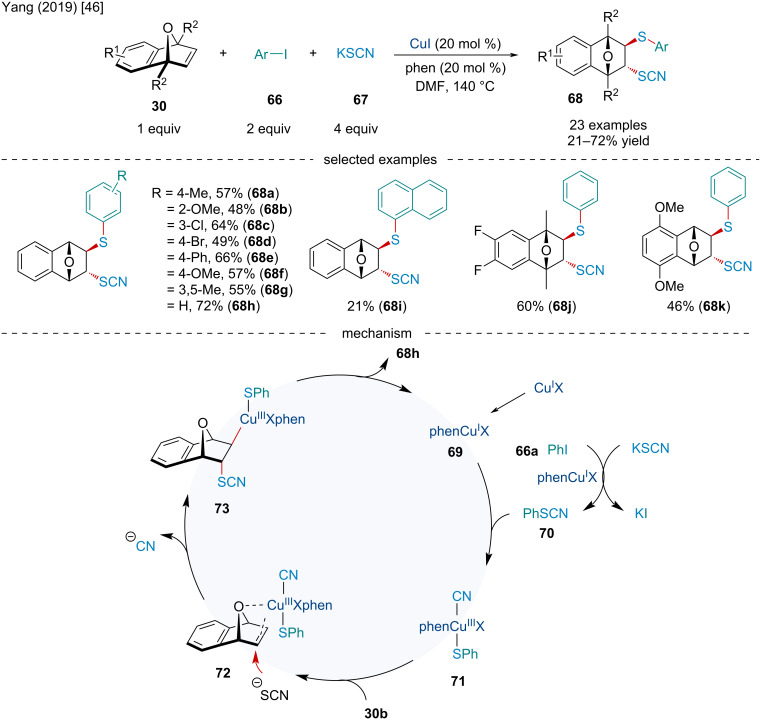
Cu-catalyzed diastereoselective 1,2-difunctionalization of oxabenzonorbornadienes **30** for the synthesis of β-thiocyanato thioethers **68**.

In 2019, the Yang lab examined the Cu-catalyzed diastereoselective 1,2-difunctionalization of oxabenzonorbornadienes **30** for the synthesis of β-thiocyanato thioethers **68** (Scheme 12) [46]. In contrast to the previous difunctionalization reactions, the authors noted the reaction was stereoselective for the *trans*-addition product. Mechanistically, the authors proposed the reaction begins with the Cu-mediated substitution reaction of iodobenzene (**66a**) with KSCN to afford phenyl thiocyanate (**70**). The Cu complex can then undergo oxidative addition into the S–C bond of the thiocyanate **70** to afford intermediate **71** which can side-on coordinate to the *exo* face of **30b**. Subsequently, the thiocyanate attacks the olefin from the *endo* face via **72** to give complex **73**. Reductive elimination furnishes the final difunctionalized product and regenerates the active Cu(I) catalyst. The reaction was broadly successful with the steric and electronic nature of the aryl iodide having little effect on the reaction.

#### Iron-catalyzed reactions

**Scheme 13:**
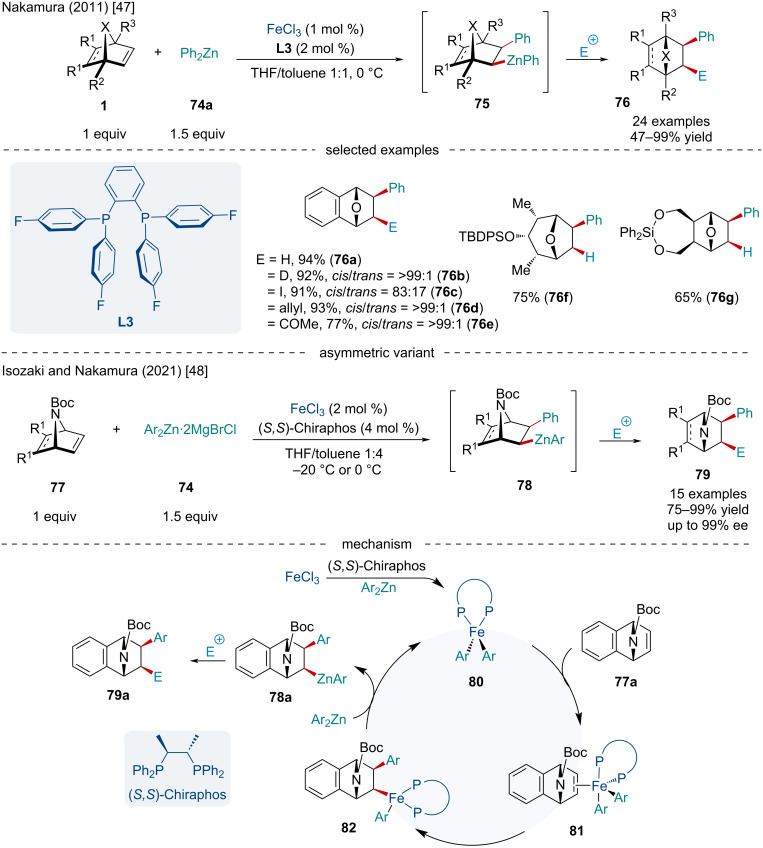
Fe-catalyzed carbozincation of heterobicyclic alkenes **1** with arylzinc reagents **74**.

Being the most earth-abundant d-block element, as well as orders of magnitude less expensive than other transition-metal catalysts, iron is bringing a renaissance to the idea of sustainable, green catalysis. In 2011, Ito et al. reported a diastereoselective Fe-catalyzed carbozincation of heterobicyclic alkenes **1** with diphenylzinc (**74a**) (Scheme 13) [47]. Using an ortho-phenylene diphosphine ligand **L3**, the authors were able to suppress β-heteroatom elimination enabling sequential electrophilic trapping of the alkylzinc complex. Although this reaction would more closely fall under the definition of a telescoped reaction than a strict domino reaction, this methodology allowed for the synthesis of difunctionalized strained alkenes. While broadly successful, strongly electron-withdrawing groups lowered the yield of the reaction. In 2021, Isozaki and Nakamura reinvestigated the reaction and established an asymmetric variant of the Fe-catalyzed carbozincation of azabicyclic alkenes **77** (Scheme 13) [48]. Using (*S,S*)-chiraphos, the authors were able to achieve enantioselectivities of up to 99%. Unfortunately, only two examples of electrophilic capturing were explored, using CD_3_CO_2_D to give deuterated products and I_2_. Most reports simply underwent protodemetalation upon quenching to afford the monosubstituted bicyclic alkene. The catalytic cycle starts with a diaryl Fe(II)–(*S,S*)-chiraphos complex **80** being generated through the reduction of FeCl_3_ with excess diarylzinc in the presence of the phosphine ligand. Side-on coordination to the *exo* face of the azabicycle **77a** generates **81** where subsequent migratory insertion affords the alkyl–Fe(II) complex **82**. Transmetalation with an organozinc produces **78a** which can be trapped by an electrophile to generate the final product **79a**.

#### Cobalt-catalyzed reactions

**Scheme 14:**
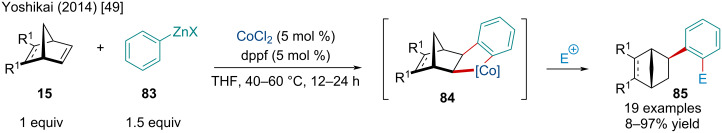
Co-catalyzed addition of arylzinc reagents of norbornene derivatives **15**.

In 2014, the Yoshikai lab investigated the Co-catalyzed addition of arylzinc reagents **83** of norbornene derivatives **15** (Scheme 14) [49]. In contrast to the 1,2-difunctionalization of bicyclic alkenes via arylzinc reagents reported by Nakamura under Fe catalysis (Scheme 13) [48], this reaction is considered to undergo a 1,4-Co migration ultimately generating 1,4-difunctionalization species. Mechanistically, the reaction likely proceeds similarly to Nakamura’s Fe-catalyzed methodology (Scheme 13) [48].

**Scheme 15:**
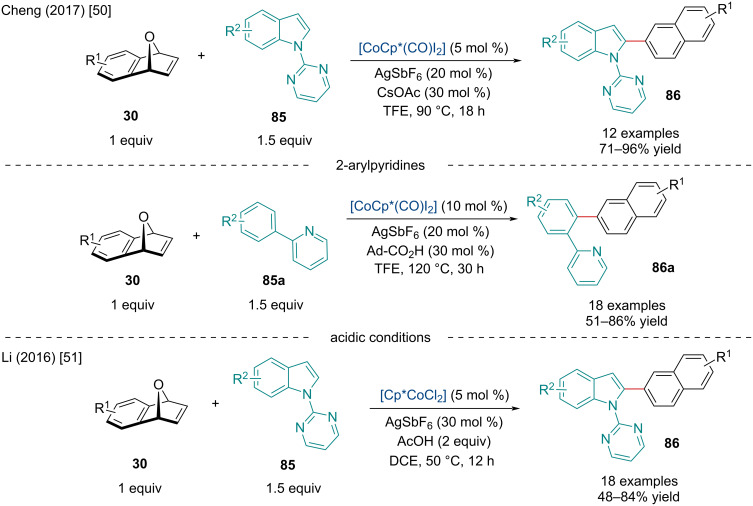
Co-catalyzed ring-opening/dehydration of oxabicyclic alkenes **30** via C–H activation of arenes.

In 2017, the Cheng laboratory investigated the Co-catalyzed ring-opening/dehydration of oxabicyclic alkenes via the C–H activation of arenes (Scheme 15) [50]. First, the group explored the *ortho*-naphthylation of *N*-pyrimidinylindole derivatives **85**. The reaction was amenable for both electron-rich and deficient indoles. When the reaction was attempted on electron-deficient oxabicyclic alkene derivatives, it was observed the reaction did not undergo dehydration to give the 2-naphthyl product, rather the ring-opened 1,2-hydroxy adduct. When the Lewis acid cocatalyst AgSbF_6_ was removed from the reaction mixture, it was noted only ring-opened 1,2-hydroxy adducts were formed, so it is likely the Lewis acid is required for dehydration. In contrast, when *N*-pyrimidinylbenzimidazole derivatives were used, the 1,2-C–H addition product was observed exclusively. By slightly altering the reaction conditions, 2-arylpyridines **85a** were able to undergo the ring-opening/dehydration reaction with oxabicyclic alkenes to afford *ortho*-naphthylated products **86a**.

Concurrently, the Li group investigated the same *ortho*-naphthylation of *N*-pyrimidinylindole derivatives **85** (Scheme 15) [51]. In contrast to Cheng’s report, it’s noted the addition of AcOH rather than CsOAc enabled the same ring-opening/dehydration cascade to occur; however, acidic conditions seem to require less energy to drive the dehydration step.

**Scheme 16:**
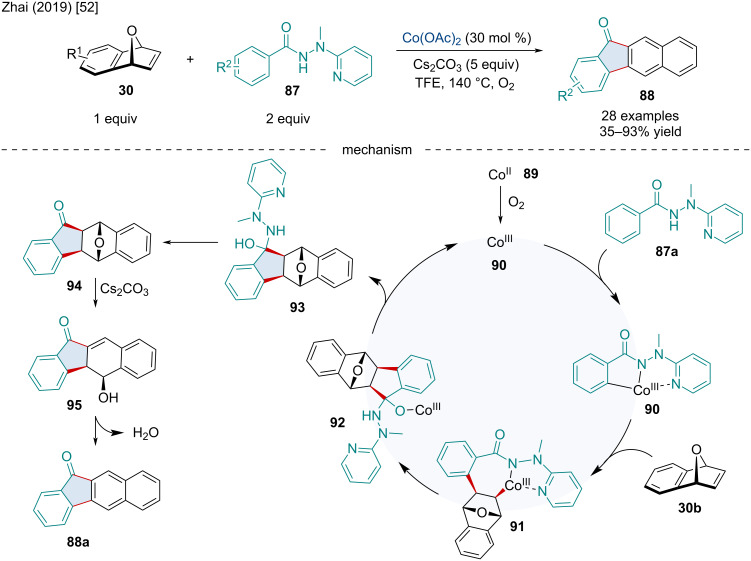
Co-catalyzed [3 + 2] annulation/ring-opening/dehydration domino reaction of oxabicyclic alkenes **1** with 2-(1-methylhydrazinyl)pyridine (MHP) directed arenes **22**.

In 2019, the Zhai Group investigated the Co-catalyzed [3 + 2] annulation/ring-opening/dehydration domino reaction of oxabicyclic alkenes **30** with 2-(1-methylhydrazinyl)pyridine (MHP) directed arenes **87** for the synthesis of benzo[*b*]fluorenones **88** (Scheme 16) [52]. C–H bond functionalization with heterobicyclic alkenes as annulation partners has received considerable attention in recent years. Several different arene and directing groups have been investigated; however, they typically result in the *exo*-selective addition product with the bridge heteroatom intact. Although this limits the applicability of the reaction, the authors noted the use of 5.0 equivalents of Cs_2_CO_3_ provided the naphthalene core via sequential dehydration. Based on preliminary mechanistic experiments, the authors proposed the reaction begins with the oxidation of Co(II) to Co(III) by O_2_. MHP-directed C–H activation of the *ortho-*C–H position generates **90** which can coordinate to the bicyclic alkene forming **91**. Migratory insertion of the olefin affords **92** which undergoes intramolecular nucleophilic addition followed by protodemetalation and elimination of MHP to afford **94**. Base-mediated ring opening of the bridging ether generates **95** which undergoes an elimination reaction to afford the naphthalene product **88a**.

**Scheme 17:**
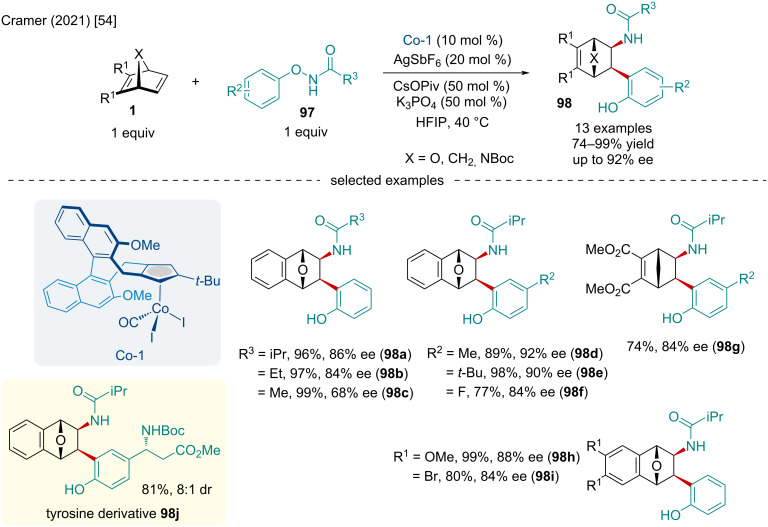
Co-catalyzed enantioselective carboamination of bicyclic alkenes **1** via C–H functionalization.

Inspired by Zhao’s seminal report on the racemic carboamination of bicyclic alkenes [53], the Cramer laboratory studied the Co-catalyzed enantioselective carboamination of bicyclic alkenes **1** via C–H functionalization in 2021 (Scheme 17) [54]. The authors noted decreasing the steric bulk of the amide moiety of the substrate from isopropyl to ethyl to methyl decreased the enantioselectivity of the reaction. Carbon- and nitrogen-bridging bicyclic alkenes were also identified as competent substrates. In this respect, norbornadiene was found to give the desired carboaminated product in slightly diminished yields while azabicyclic alkenes generated the targeted products in excellent yield, albeit with slightly reduced enantioselectivity. To showcase the synthetic capabilities of this methodology, the authors synthesized the non-natural amino acid derivative **98j** in good diastereoselectivity.

### Ruthenium-catalyzed reactions

**Scheme 18:**
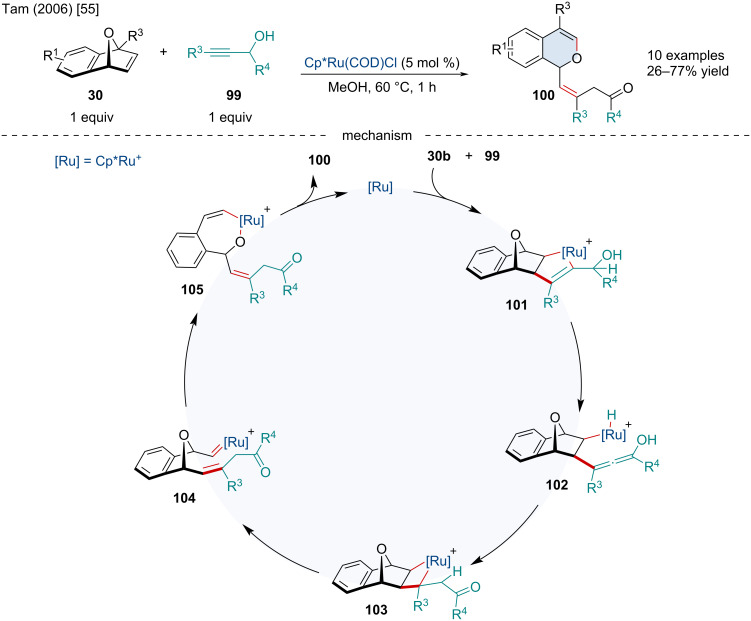
Ru-catalyzed cyclization of oxabenzonorbornene derivatives with propargylic alcohols for the synthesis of isochromenes.

In 2006, the Tam laboratory investigated the Ru-catalyzed cyclization of oxabenzonorbornene derivatives **30** with propargylic alcohols **99** for the synthesis of isochromenes **100** (Scheme 18) [55]. After coordination of the Ru-center to the *exo* face of **30b**, oxidative cyclization can afford the ruthenacycle **101**. Unlike previous works studying Ru-catalyzed cyclizations involving bicyclic alkenes and alkynes [56–59], the reaction preferentially undergoes β-hydride elimination to generate **102** rather than reductive elimination which would afford the [2 + 2] adduct. Hydroruthenation of the allene produces **103** which can either undergo reductive elimination to afford the cyclopropanated bicyclic alkene or undergo a [2 + 2] cycloreversion to generate the Ru–carbene **104**. The Ru–carbene **104** can rearrange to **100** through a 1,3-migration of the alkoxy group which can finally reductively eliminate the isochromene product. Based on control reactions, the authors proposed the active catalytic species is cationic, as the use of the cationic precatalyst [Cp*Ru(CH_3_CN)_3_]PF_6_ in THF afforded the isochromene as the major product, suggesting a similar cationic species may be generated in MeOH [60].

**Scheme 19:**
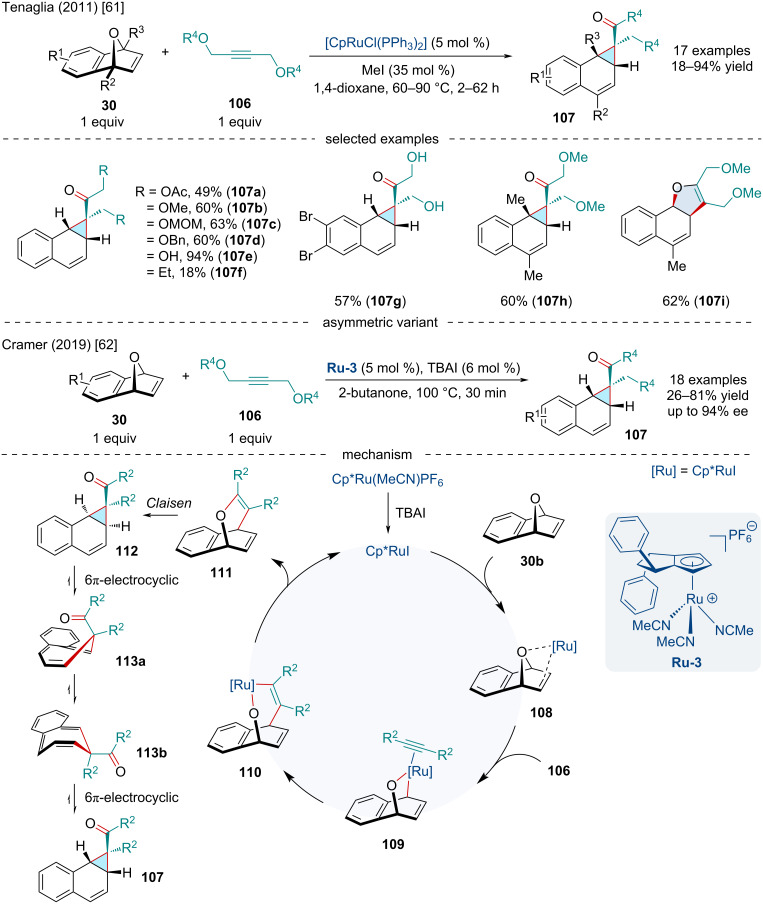
Ru-catalyzed coupling of oxabenzonorbornene derivatives **30** with propargylic alcohols and ethers **106** to access benzonorcaradienes **107**.

In 2011, Tenaglia and co-workers investigated the Ru-catalyzed coupling of oxabenzonorbornene derivatives **30** with propargylic alcohols and ethers **106** to access benzonorcaradienes **107** (Scheme 19) [61]. While discriminating between the neutral and cationic active ruthenium species, the authors noted the use of [Cp*Ru(CH_3_CN)_3_]PF_6_ as the precatalyst produced the cyclopropanated bicyclic alkene adducts exclusively. This contrasts with Tam’s report (Scheme 18) [55] which found cationic Ru species formed the isochromene **100** preferentially which may be attributed to the solvent playing a more impactful role in the reaction than previously anticipated. Of note, the reaction was amenable to a broad scope of derivatized heterobicyclic alkenes. Electron-deficient bicyclic alkenes were found to react much slower, ultimately affording products in diminished yields. Mono- and disubstituted bridgehead variants were applicable, but with reduced efficacy with the former producing a dihydronaphthofuran **107i** as the major product.

In 2019, the Cramer group continued studying this reaction and developed an enantioselective variant utilizing a chiral Cp* derivative (Scheme 19) [62]. Similar reactivity trends were observed in both accounts. Mechanistically, the transformation was proposed to begin with the coordination of Cp*RuI to the *exo* face of the bicyclic alkene. Oxidative addition into the C–O bond, which is proposed to be the enantiodetermining transition state, followed by coordination to the alkyne generates intermediate **109**. Migratory insertion of the alkyne results in the ruthenacycle **110**. Subsequent reductive elimination generates putative allyl vinyl ether **111** and regenerates the active ruthenium complex**.** The allyl vinyl ether intermediate undergoes a Claisen rearrangement to afford the *endo*-isomer **112**. Thermal isomerization of **113a** by a 6π-electrocyclic ring-opening/closing cascade leads to the to the final *exo*-isomer **107**.

**Scheme 20:**
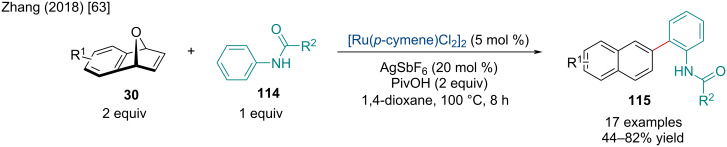
Ru-catalyzed ring-opening/dehydration of oxabicyclic alkenes via the C–H activation of anilides.

In 2018, the Zhang lab investigated the Ru-catalyzed ring-opening/dehydration of oxabicyclic alkenes **30** via the C–H activation of anilides **114** (Scheme 20) [63]. When the optimized conditions were applied to azabenzonorbornadiene derivatives, the dehydrative naphthylation sequence did not occur with the reaction being exclusive for *exo*-ring-opened products, similar to that observed in a typical Rh-catalyzed ring-opening reaction (vide infra). The reaction seems to be sensitive to the steric bulk of the amide functionality with *n*-propyl and isopropylamides having diminished yields. While the scope of anilides was quite extensive, electron-deficient substrates resulted in lowered yields.

**Scheme 21:**
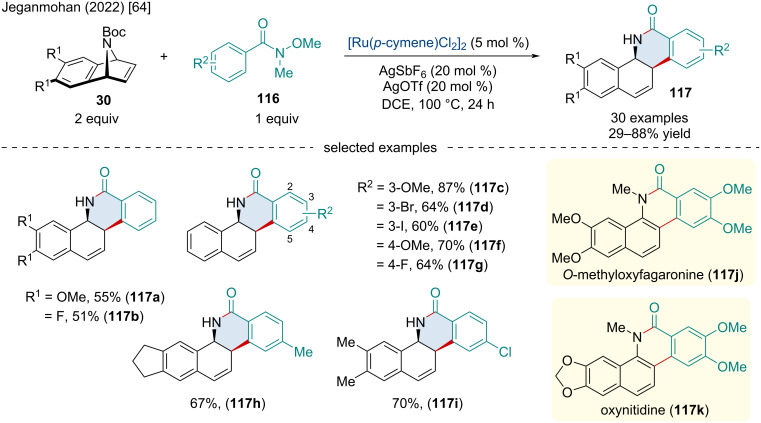
Ru-catalyzed of azabenzonorbornadiene derivatives with arylamides.

In 2022, the Jeganmohan group investigated the Ru-catalyzed ring-opening/lactamization of azabenzonorbornadiene derivatives **30** with arylamides **116** (Scheme 21) [64]. Weinreb amides outperformed other arylamides, likely serving as a better directing group for the initial aryl-C–H activation. While the scope of functionalized aryl Weinreb amides was quite wide, including different EWGs and EDGs, as well as heterocycles, *ortho*-substitution was not tolerated. The authors applied the methodology for the synthesis of biologically important benzo[*c*]phenanthridine derivatives **117**. Through methylation and subsequent aromatization of the phenanthridinones produced, the authors were able to quickly afford novel fagaronine **117j** and nitidine **117k** derivatives.

### Rhodium-catalyzed reactions

**Scheme 22:**
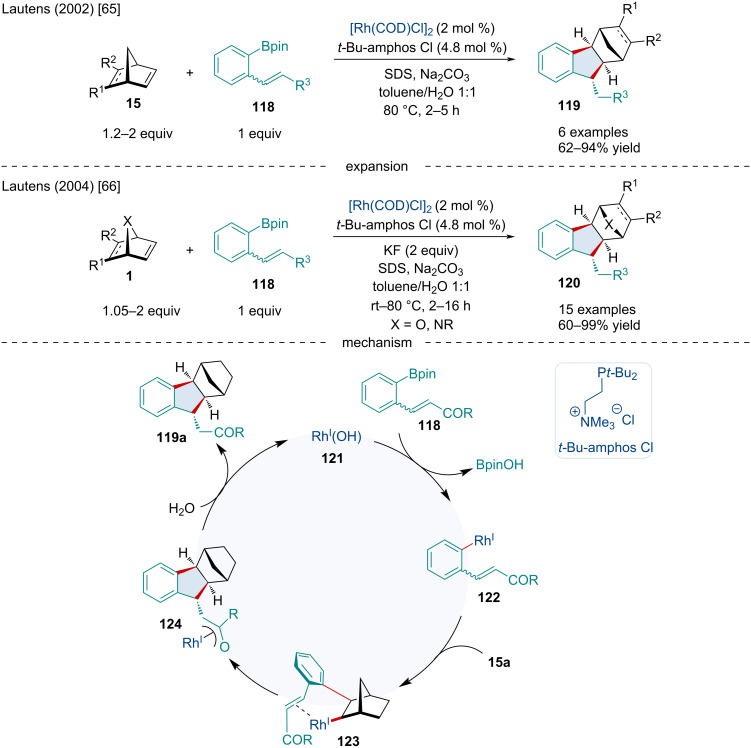
Rh-catalyzed cyclization of bicyclic alkenes with arylboronate esters **118**.

In 2002, the Lautens laboratory reported a tandem cyclization of arylboronate esters **118** with a variety of bicyclic alkenes **15** using a water-soluble Rh-catalytic system (Scheme 22) [65]. The authors reported the reaction proceeded smoothly with a limited variety of substituted norbornenes and boronate esters.

In 2004, the same group expanded this Rh-catalyzed cyclization to heterobicyclic alkenes **1** with arylboronate esters **118** for the synthesis of a variety of functionalized indanes **120** (Scheme 22) [66]. This reaction proceeded smoothly with a broad range of [2.2.1] and [3.2.1]-bicyclic alkenes; however, doubly bridgehead-substituted bicyclic alkenes exclusively produced an undesirable demetalated aryl ester byproduct. The authors attributed this to a steric prevention of the attack of the arylrhodium nucleophile to the alkene. Azabicyclic alkenes also proved difficult and failed to react. Mechanistically the authors proposed the arylboronate ester **118** first undergoes a transmetalation with the Rh(I) complex producing **122** which performs an *exo*-carborhodation with the bicyclic substrate to produce **123**. A 5-*exo*-*trig* ring closure of **123** produces **124** followed by a rapid protodemetalation with water releasing the final indane product **119a** and regenerating the active Rh(I) species. The authors proposed that the origin of the diastereoselectivity is due to significant steric interactions between the –COR group on the pendant alkene and the bridging group of the bicyclic alkene in **123**.

**Scheme 23:**
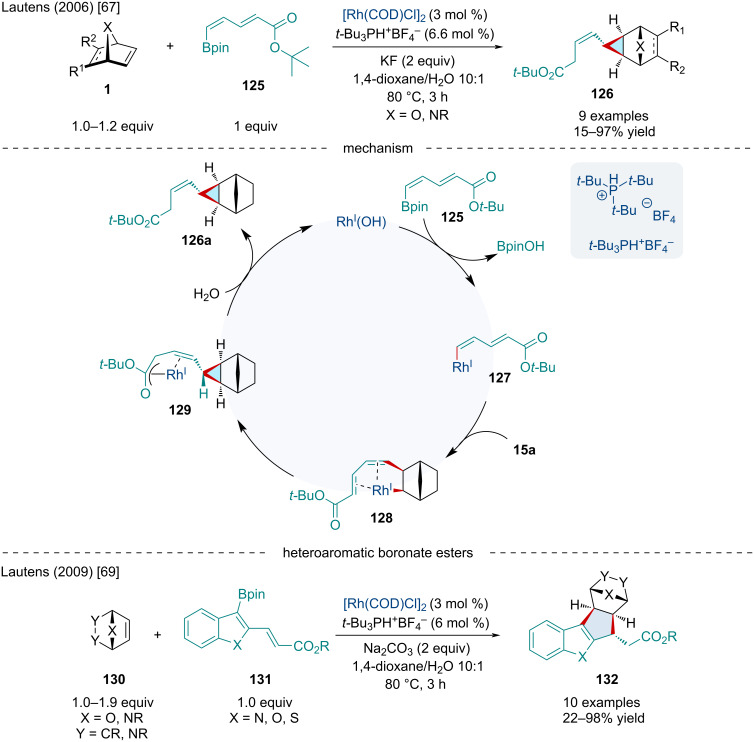
Rh-catalyzed cyclization of bicyclic alkenes with dienyl- and heteroaromatic boronate esters.

In 2006, the Lautens lab continued to extend this reaction to include dienylboronate esters **125** and found that an unexpected vinylcyclopropane product **126** was generated (Scheme 23) [67]. Again, nitrogen-containing bicyclic alkenes proved difficult, as diazabicyclic alkenes produced the desired product in low yields while azabenzonorbornadienes failed to react entirely. It was found the introduction of a methyl group α to the boron on the dienylboronate caused the selectivity to be shifted to the 1,4-addition producing a cyclopentene product leading to the conclusion that the substitution pattern on the boronate ester played a significant role in the selectivity between 1,6-addition and 1,4-addition. The mechanism proposed by the authors initially begins in the same manner as Scheme 22 with the transmetalation of the boronate ester with Rh(I) producing **127** which undergoes an *exo*-carborhodation with the bicyclic substrate **15a** producing **128**. The reaction path diverges from the previous mechanism undergoing a 1,6-addition resulting in **129**. A rapid protodemetalation with water then occurs releasing the final vinylcyclopropane product **126a** and regenerating the active Rh(I) species. A later 2009 investigation revealed methyl groups α to the ester produced a hydrofunctionalization product [68]. Dienylboronate esters bearing methyl groups β to the ester group produced vinylcyclopropane products **126** while dienylboronate esters bearing methyl groups at the δ or γ position resulted in cyclopentene products.

In 2009, the Lautens lab continued investigating the reactions of bicyclic alkenes **130** with a novel range of heteroaromatic boronate esters **131** (Scheme 23) [69]. This has previously been a challenging transformation due to the propensity of these systems to produce non-cyclized hydroarylation products due to an unproductive rhodium 1,4-migration on heteroaromatic moieties. The use of benzothiophene, benzofurans, and benzopyrrole boronate esters in this investigation prevented this shift as these systems lack the hydrogen to participate in this shift. This reaction proceeded smoothly with a variety of bicyclic alkenes although diazabicyclic alkenes had little to no reactivity. Moreover, benzofuran and benzopyrrole boronate esters resulted in low yields.

**Scheme 24:**
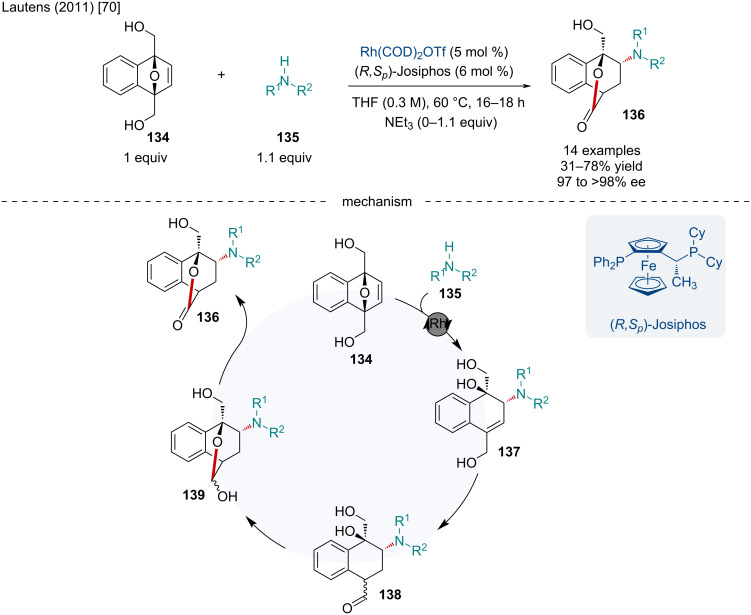
Rh-catalyzed domino lactonization of doubly bridgehead-substituted oxabicyclic alkenes with secondary amine nucleophiles.

In 2011, the Lautens lab reported the Rh-catalyzed domino reaction of doubly bridgehead-substituted oxabicyclic alkenes **134** with secondary amine nucleophiles **135** for the synthesis of bicyclo[2.2.2]lactones **136** (Scheme 24) [70]. This reaction proceeded smoothly with a variety of secondary amine nucleophiles, including those with hydrocarbon, ether, acetal, and ester functionalities; although, aniline nucleophiles only resulted in the one step asymmetric ring-opening (ARO) product under the standard reaction conditions. Fortunately, the authors noted the addition of triethylamine allowed for aniline nucleophiles to undergo the domino reaction, generating the desired bicyclo[2.2.2]lactone **136**. The authors proposed the reaction first takes place through an ARO of the doubly bridgehead-substituted oxabicyclic alkene with the secondary amine nucleophile ultimately producing **137**. The Rh(I) catalyst then facilitates the allylic alcohol isomerization in **137** resulting in the aldehyde **138**. This aldehyde, in close proximity to the tertiary alcohol, leads to the production of the hemiacetal **139** which can finally undergo an oxidation producing the final bicyclo[2.2.2]lactone product **136**.

**Scheme 25:**
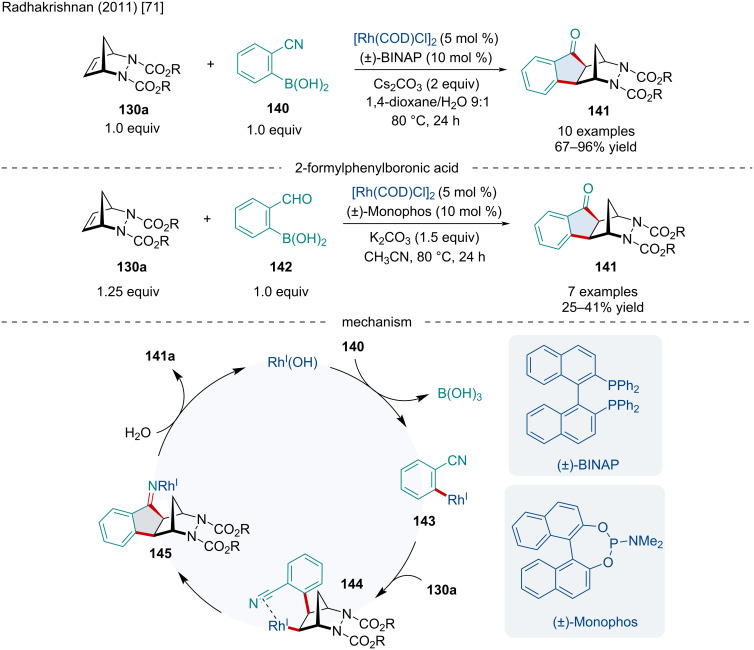
Rh-catalyzed domino carboannulation of diazabicyclic alkenes with 2-cyanophenylboronic acid and 2-formylphenylboronic acid.

In 2011, the Radhakrishnan laboratory reported the carboannulation of diazabicyclic alkenes **130a** with 2-cyanophenylboronic acid (**140**) and 2-formylphenylboronic acid (**142**) for the synthesis of indanones **141** (Scheme 25) [71]. This reaction proceeded smoothly with a variety of substituted diazabicyclic alkenes including a variety of ester substituents on the nitrogens and sterically more hindered tricyclic adducts. Mechanistically, the authors proposed the reaction begins with a transmetalation of 2-cyanophenylboronic acid with the Rh(I) species resulting in **143**. Upon association of **143** with the diazabicyclic alkene **132a** a *syn exo*-addition occurs producing **144**. Subsequently, coordination of the Rh(I) to the electrophilic cyano group leads to an intramolecular addition producing **145**. The imine undergoes a hydrolysis releasing the final carboannulated product **141** as well as regeneration of the active Rh(I) catalyst. A similar mechanism can be envisioned for the carbonannulation of diazabicyclic alkenes with 2-formylphenylboronic acid up to the last step which likely operates through a β-hydride elimination of the Rh(I) alkoxide, furnishing the final carbonyl-containing product.

**Scheme 26:**
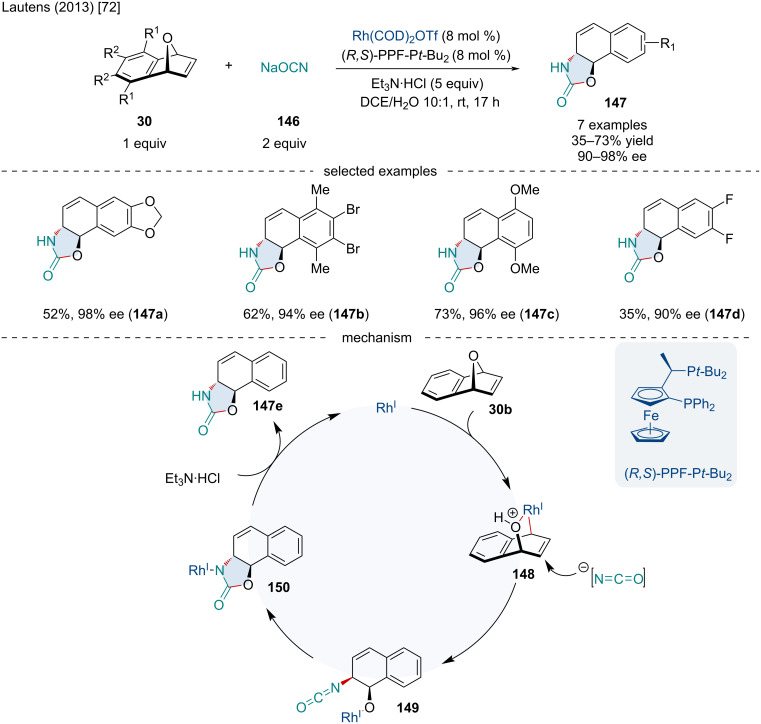
Rh-catalyzed synthesis of oxazolidinone scaffolds **147** through a domino ARO/cyclization of oxabicyclic alkenes **30** with sodium cyanate (**146**).

In 2013, Lautens reported the synthesis of oxazolidinone scaffolds **147** through a domino ARO reaction followed by cyclization reaction of oxabicyclic alkenes **30** with sodium cyanate (**146**) (Scheme 26) [72]. This reaction proceeded smoothly with electron-rich oxabenzonorbornadiene derivatives; however, electron-poor oxabenzonorbornadiene derivatives resulted in reduced yield and enantioselectivity. Bridgehead-substituted, non-benzo-fused oxabicycles, as well as azabicyclic alkenes failed to produce the desired product. When the benzo-fused moiety was unsymmetrically substituted, little regioselectivity was observed. Based on X-ray crystallographic data for their final product, and previously reported Rh-catalyzed ARO reactions, the authors hypothesized the reaction begins with the oxidative addition of the Rh(I) catalyst into the bridgehead C–O bond of the oxabenzonorbornadiene producing **148** which is considered the enantiodetermining step. The isocyanate anion then nucleophillically attacks the alkene in an S_N_2’ fashion producing the *trans*-isocyanate **149**. Subsequently, insertion of the Rh–O bond into the isocyanate results in **150**. Finally, protonolysis produces the oxazolidinone product **147e** as well as regenerates the active Rh(I) catalyst.

**Scheme 27:**
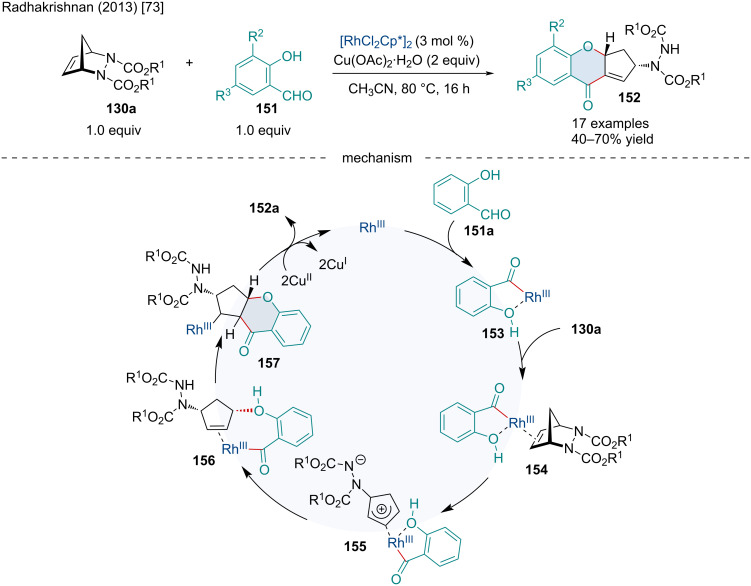
Rh-catalyzed oxidative coupling of salicylaldehyde derivatives **151** with diazabicyclic alkenes **130a**.

In 2013, the Radhakrishnan laboratory reported the Rh-catalyzed oxidative coupling of salicylaldehyde derivatives **151** with diazabicyclic alkenes **130a** producing fused chromanone derivatives **152** (Scheme 27) [73]. It was determined alkyl- and methoxy-substituted salicylaldehydes resulted in a minor reduction of yield while salicylaldehydes with EWGs failed to react. The authors hypothesized the reaction mechanism begins with the association of the Rh(III) catalyst with the hydroxy group of salicylaldehyde (**151a**) resulting in a selective cleavage of the aldehyde C–H bond producing the rhodocycle **153** which side-on coordinates with the alkene of the azabicycle producing **154**. A C–N bond cleavage occurs creating π-allylrhodium **155**. Subsequently, the phenol oxygen then adds to the π–allyl species in a *cis* fashion, furnishing **156** which is proposed to be the enantiodetermining step. The carbonyl–rhodium species **156** inserts into the alkene to produce **157**. Following this, β-hydride elimination occurs yielding the final product **152** and a Rh(I) species which is oxidized back to its active Rh(III) state by Cu(OAc)_2_·H_2_O.

**Scheme 28:**
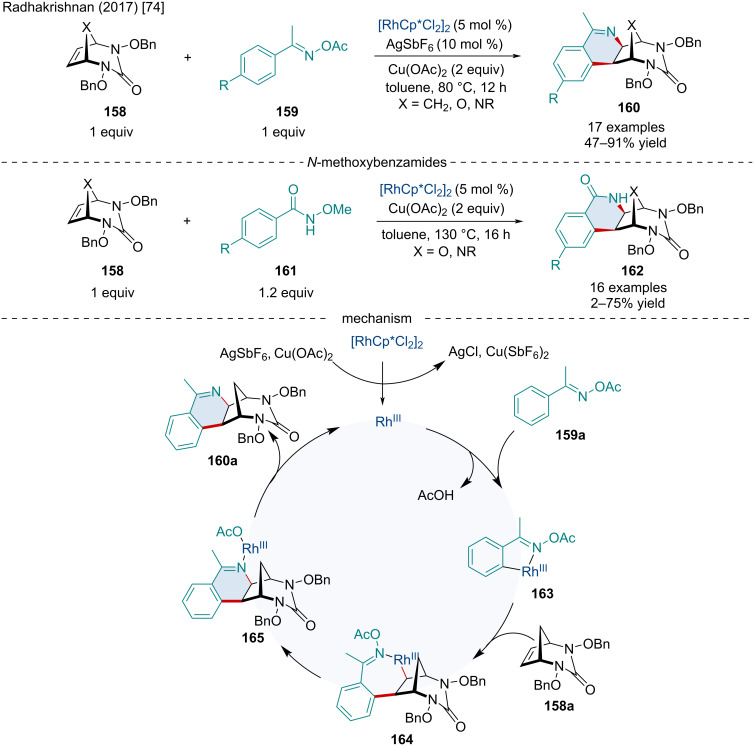
Rh-catalyzed reaction of *O*-acetyl ketoximes with bicyclic alkenes for the synthesis of isoquinoline-fused bicycles.

In 2017, Radhakrishnan reported a Rh-catalyzed annulation of *O*-acetyl ketoximes **159** or *N*-methoxybenzamides **161** with [2.3.1]-bicyclic alkenes **158** for the synthesis of isoquinoline (**160**) or isoquinolone-fused bicycles **162** (Scheme 28) [74]. Compared to their previous C–H functionalization reaction (Scheme 27) [73], no ring opening was observed. This reaction with *O*-acetyl ketoximes was amenable to a variety of *para-*substituents including methoxy and halide groups; however, *O*-acetyl ketoximes with *ortho-* or *meta*-substituents failed to react. A small number of substituted [2.2.1]diazabicyclic alkenes **130a** were successfully employed, albeit with slightly lower yields. In the reaction with *N*-methoxybenzamides **161**, the same substituent trends were seen as that with the reaction with *O*-acetyl ketoximes. Mechanistically, the reaction begins when the Rh(III) catalyst is converted to an active Rh(III) species, by AgSbF_6_ and Cu(OAc)_2_, which oxidatively inserts into the *ortho* C–H bond forming **163**. Migratory insertion of the alkene forms **164**. Next, cleavage of the N–O bond followed by an oxidative addition of the Rh(III) to the N–O bond forms intermediate **165** which can finally undergo reductive elimination giving the final product **160a**.

**Scheme 29:**
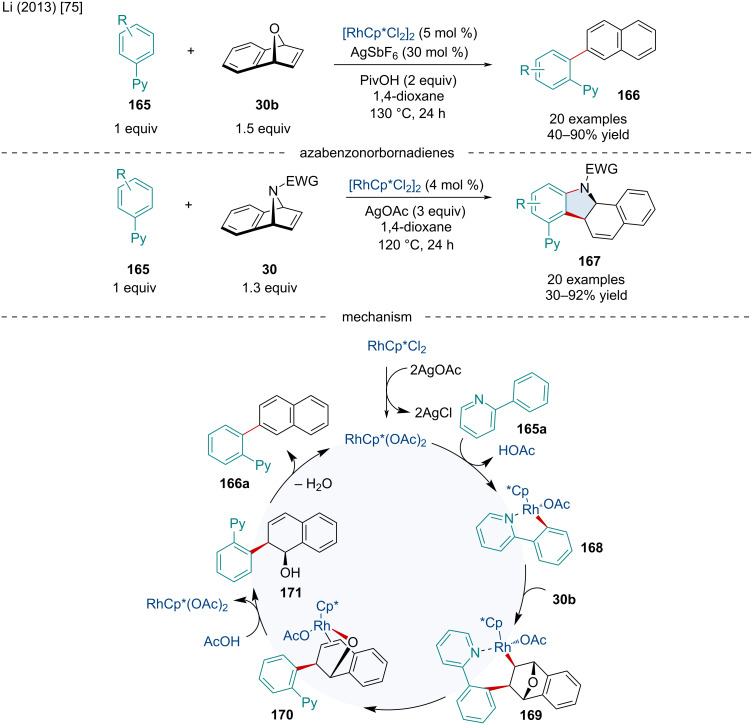
Rh-catalyzed domino coupling reaction of 2-phenylpyridines **165** with oxa- and azabicyclic alkenes **30**.

In 2013, Li reported the domino coupling reaction of 2-phenylpyridines **165** with oxa- and azabicyclic alkenes **30** (Scheme 29) [75]. When investigating the reaction with oxabenzonorbornadiene (**30b**), the resulting product was found to exclusively be the dehydrated 2-naphthalene derivative **166**. It was found that the addition of pivalic acid greatly improved the yield, likely due to its facilitation of C–H activation as well as its involvement in the dehydration process. This reaction proceeded smoothly with a variety of both EWGs and EDGs on the 2-phenylpyridine. Interestingly, when swapping the pyridine directing group for thiophene or furan, yields were improved although quinolinyl and pyrimidyl directing groups, despite reacting, resulted in a mixture of mono- and diarylation products. When investigating substituted oxabenzonorbornadienes both mono- and diarylated products were formed with only moderate yield. When azabenzonorbornadienes **30** were investigated in the same redox-neutral conditions no reaction occurred; however, upon the addition of AgOAc a *cis*-fused dihydrocarbazole product was formed (Scheme 29). Mechanistically this reaction was proposed to proceed through first a conversion of the Rh(III) catalyst to the active Rh(III) species by AgSbF_6_. This active Rh(III) catalyzes the cleavage of the *ortho*-C–H bond of 2-phenylpyridine furnishing **168**. This is followed by the *cis* addition of **168** to the oxabenzonorbornadiene producing **169** whereby subsequent β-oxygen elimination affords **170**, followed by protonolysis producing **171** and regenerating the active Rh(III) species. Finally, a dehydration occurs furnishing the final product **166**. In terms of the azabicyclic substrates, following the β-eliminated heteroatom, a second round of C–H activation/reductive elimination occurs to generate the annulated product **167**.

**Scheme 30:**
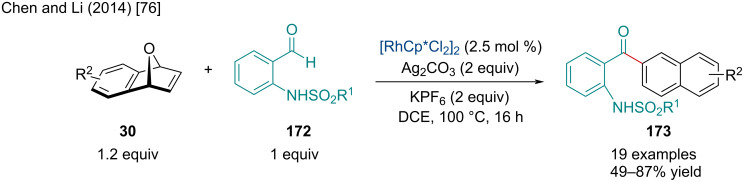
Rh-catalyzed domino dehydrative naphthylation of oxabenzonorbornadienes **30** with *N*-sulfonyl 2-aminobenzaldehydes **172**.

In 2014, Chen and Li reported the Rh-catalyzed domino dehydrative naphthylation of oxabenzonorbornadienes **30** with *N*-sulfonyl 2-aminobenzaldehydes **172** (Scheme 30) [76]. This reaction was amenable to a variety of EDG, EWG, as well as a broad scope of sulfonyl groups. Surprisingly, this reaction also proceeded smoothly with nitro substituents on the benzene ring which are typically problematic in C–H activation reactions. Through mechanistic studies, the authors proposed the rate limiting step for this reaction is the C–H cleavage.

**Scheme 31:**
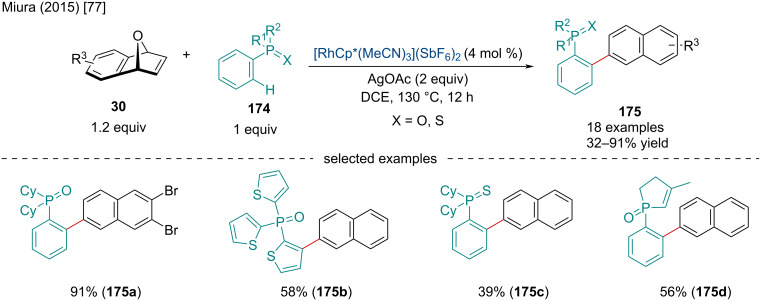
Rh-catalyzed domino dehydrative naphthylation of oxabenzonorbornadienes **30** with arylphosphine derivatives **174**.

In 2015, Miura and co-workers reported the Rh-catalyzed domino dehydrative naphthylation of oxabenzonorbornadienes **30** with arylphosphine derivatives **174** (Scheme 31) [77]. The reaction was amenable to a wide range of substituted arylphosphine derivatives. Moreover, the reaction could be extended to include various phosphinate, phosphonate, and phosphonamide derivatives. The use of triarylphosphine oxides required the reaction to be performed at a 2:1 ratio with oxabenzonorbornadienes **30** to prevent multiarylated products from being formed. Arylphosphine sulfides were also investigated but gave unimpressive yields (8%); however, upon a substitution of the AgOAc for 3 equiv of AcOH moderate yields were obtained (39%). Mechanistically, this reaction likely operates in a similar manner to the previously discussed C–H activation/dehydration domino reactions.

In 2020, a similar method for the construction of 2-aryl-substituted naphthalene cores was discussed by Wang and co-workers who disclosed a Rh-catalyzed C–H bond naphthylation of anilides and benzamides with azabenzonorbornadienes [78]. Interestingly, the dehydration step occurred smoothly with an aza-leaving group rather than the more common oxa-leaving group discussed above.

**Scheme 32:**
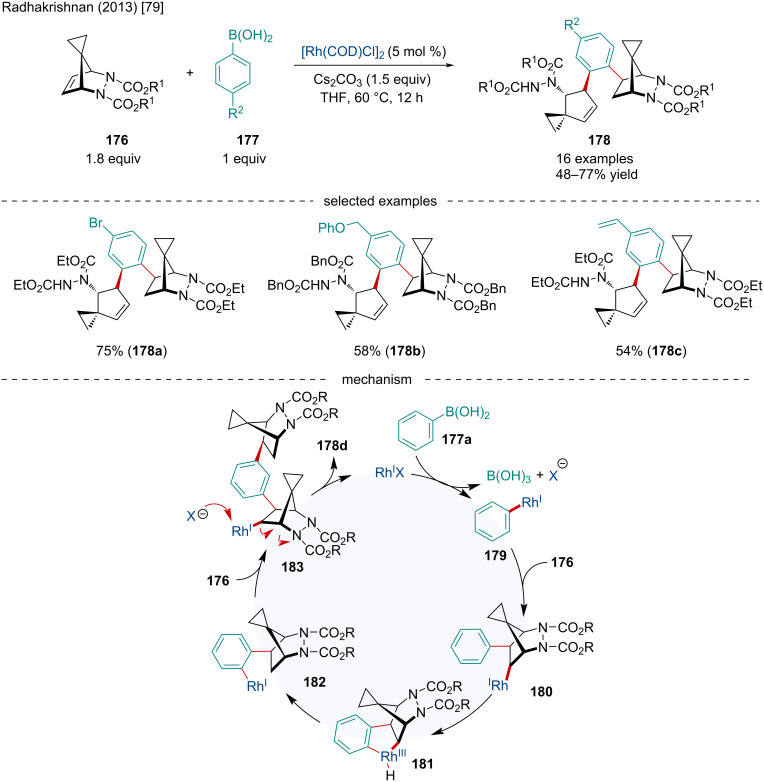
Rh-catalyzed domino ring-opening coupling reaction of azaspirotricyclic alkenes using arylboronic acids.

In 2013, the Radhakrishnan laboratory reported the Rh-catalyzed domino ring-opening coupling reaction of azaspirotricyclic alkenes **176** using arylboronic acids **177** (Scheme 32) [79]. This reaction proceeded well with a variety of ester substituents on the nitrogens of the azaspirotricyclic alkenes. The authors proposed this reaction proceeds first through a transmetalation of the arylboronic acid **177a** with the Rh(I) catalyst producing **179** which undergoes a *cis* addition to the azaspirotricyclic alkene resulting in intermediate **180**. C–H cleavage at the *ortho-*position followed by an intramolecular reductive elimination affords in **182**. Unlike previous reports [80], this arylrhodium complex has a long enough lifetime to propagate further. A subsequent migratory insertion into a second azaspirotricyclic alkene furnishes **183**. Finally, the anion from the catalyst attacks **183** causing a ring opening, forming the final product **178d** and regenerating the Rh(I) catalyst. Keeping with other mechanisms, the Rh(I) may also undergo an *anti*-β-nitrogen elimination to furnish the ring-opened intermediate [80].

**Scheme 33:**
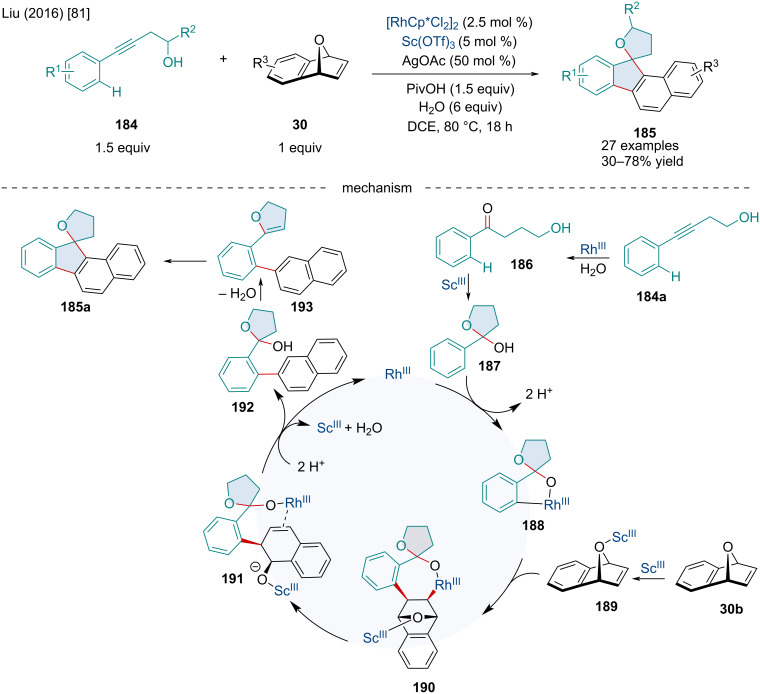
Tandem Rh(III)/Sc(III)-catalyzed domino reaction of oxabenzonorbornadienes **30** with alkynols **184** directed by a transient hemiketal group.

In 2016, Liu reported the Rh(III)/Sc(III)-catalyzed domino reaction of oxabenzonorbornadienes **30** with alkynols **184** directed by a transient hemiketal group (Scheme 33) [81]. The use of a transient directing group avoids the tedious process of installation and then removal of directing groups which is a common issue. A variety of substituents were tolerated on both the alkynols **184** and oxabenzonorbornadienes **30**; however, substituted oxabenzonorbornadiene derivatives typically had diminished reactivity. Expansion of the bicyclic scope was limited as other bicycles such as norbornene failed to react. The authors propose the catalytic cycle begins with the Rh(III)-catalyzed hydration of the alkynol to produce **186** followed by a Sc(III)-catalyzed addition to form the transient hemiketal **187**. *Ortho*-C–H activation generates **188** which can undergo migratory insertion with the Sc(III)-coordinated oxabicyclic alkene **189** to form **190**. β-Oxygen elimination, likely assisted by the Sc(III) Lewis acid, produces **191** which subsequently undergoes a protonolysis forming **192** and regenerating the Rh(III) and Sc(III) catalysts. Next, **192** is dehydrated producing **193** which finally undergoes a Prins-type cyclization to afford the final product **185**.

**Scheme 34:**
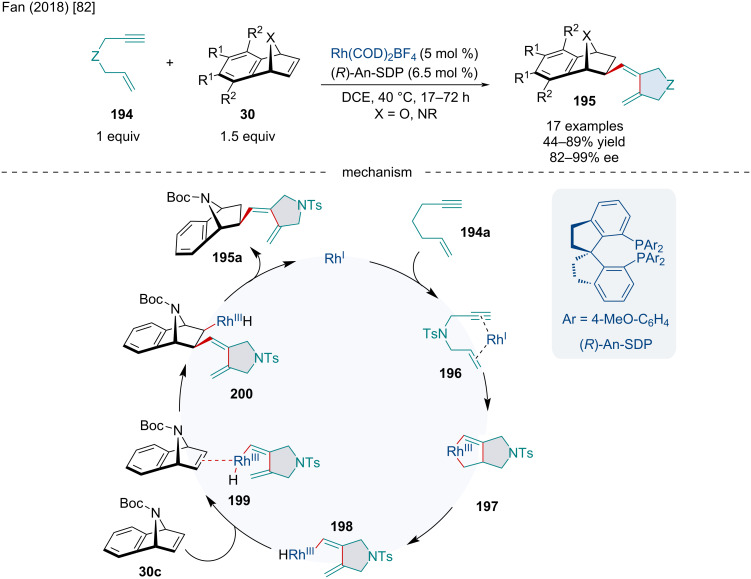
Rh-catalyzed asymmetric domino cyclization and addition reaction of 1,6-enynes **194** and oxa/azabenzonorbornadienes **30**.

In 2018, the Fan laboratory reported the Rh-catalyzed asymmetric cyclization/addition domino reaction of 1,6-enynes **194** with oxa/azabenzonorbornadienes **30** (Scheme 34) [82]. Both oxa- and azabenzonorbornadienes **30** worked well; however, the authors noted the latter produced better enantioselectivities while sterically bulky substituents led to both reduced yield and enantioselectivities. The authors proposed the reaction mechanistically occurs though the coordination and reaction of the Rh(I) species with the 1,6-enyne **194a** producing **196** which undergoes an oxidative cyclization leading to **197**. Subsequent β-hydride elimination forms **198** which side-on coordinates with azabenzonorbornadiene **30c** forming **199**. Migratory insertion of the olefin followed by reductive elimination of the hydride affords the final product **195a**.

**Scheme 35:**
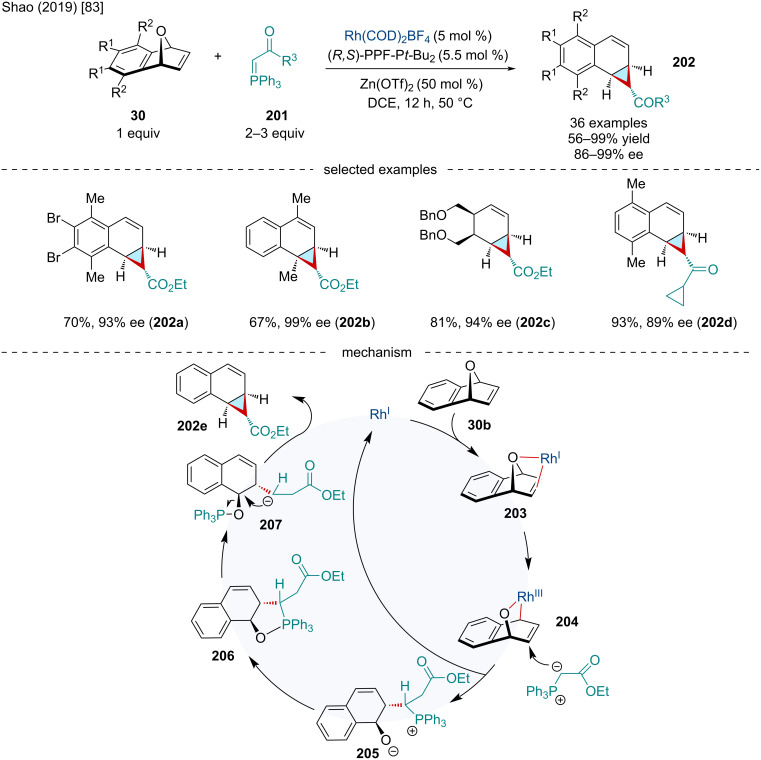
Rh/Zn-catalyzed domino ARO/cyclization of oxabenzonorbornadienes **30** with phosphorus ylides **201**.

In 2019, the Shao group reported the Rh/Zn-catalyzed domino ARO/cyclization of oxabenzonorbornadienes **30** with phosphorus ylides **201** (Scheme 35) [83]. Despite the difficulty of using phosphorus ylides as nucleophiles in metal-catalyzed reactions due to their ability to strongly bind transition metals, this reaction proceeded smoothly with a broad range of ester-, ketone-, and amide-stabilized phosphorus ylides. Oxabenzonorbornadienes bearing both EWG and EDG substituents worked well including bridgehead-substituted substrates which only experienced a slight reduction in yield. Similar to other ARO reactions, the catalytic cycle is proposed to begin with the oxidative insertion of the Rh(I) catalyst into the bridgehead C–O bond producing **204**. The phosphorus ylide attacks **204** in an S_N_2’ fashion on the *endo* face giving the ring-opened **205** as well as regenerating the Rh(I) catalyst after dissociation. Alternatively, **205** can undergo a ring closure followed by a subsequent C–P-bond cleavage causing a ring opening resulting in **207**. Intramolecular S_N_2’ and elimination of the phosphine oxide generates the final product **202e** which the authors propose is stereoselective due to significant steric interactions between the carbonyl and aryl groups. The authors proposed that the Zn(OTf)_2_ Lewis acid cocatalyst may activate the bridging oxygen of the oxabenzonorbornadiene lowering the kinetic barrier of C–O oxidative addition.

**Scheme 36:**
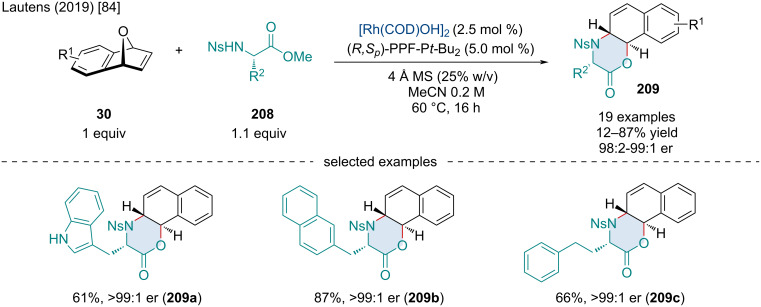
Rh-catalyzed domino ring opening/lactonization of oxabenzonorbornadienes **30** with 2-nitrobenzenesulfonamides amino acid-derived nucleophiles **208**.

In 2019, Lautens reported the Rh-catalyzed domino ring opening/lactonization of oxabenzonorbornadienes **30** with nosyl-protected amino acid-derived nucleophiles **208** (Scheme 36) [84]. This reaction proceeded smoothly with a range of amino acid derivatives; however, the authors noted that increased steric bulk of the nucleophiles reduced the yields which they attributed to the lactonization being disfavored on steric grounds. In contrast to other ARO reactions, substituents on the oxabicycles were not tolerated well and only two derivatized substrates successfully reacted with greatly diminished yields. Moreover, amino acid derivatives without α-substituents failed to react, leading the authors to conclude that α-substitution is required to make lactonization kinetically feasible.

**Scheme 37:**
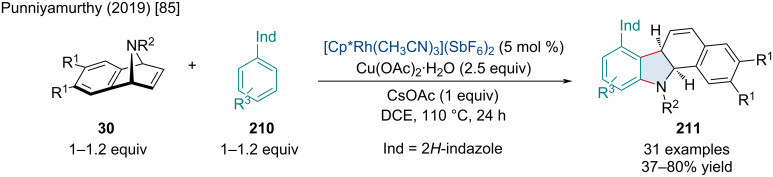
Rh-catalyzed domino C–C/C–N bond formation of azabenzonorbornadienes **30** with aryl-2*H*-indazoles **210**.

In 2019, the Punniyamurthy lab reported the Rh-catalyzed domino C–C/C–N bond formation of azabenzonorbornadienes **30** with aryl-2*H*-indazoles **210** (Scheme 37) [85]. This reaction was amenable to both EWGs and EDGs; however, it was noted that an azabenzonorbornadiene bearing a pyridine-2-sulfonyl protecting group only produced a trace amount of product which was attributed by the authors to an unproductive chelation of the Rh(III) by the pyridine nitrogen. Furthermore, aryl-2*H*-indazoles with *para*-substituents failed to react which the authors attributed to both electronic and steric effects.

**Scheme 38:**
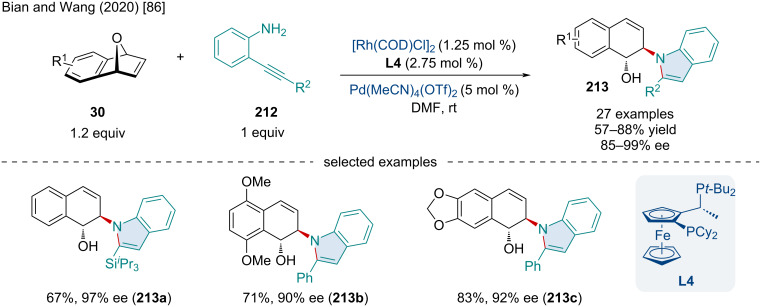
Rh/Pd-catalyzed domino synthesis of indole derivatives with 2-(phenylethynyl)anilines **212** and oxabenzonorbornadienes **30**.

In 2020, Bian and Wang reported the Rh/Pd-catalyzed domino reaction of indole derivatives with 2-(phenylethynyl)anilines **212** and oxabenzonorbornadienes **30** (Scheme 38) [86]. In this reaction, both EWG and EDG substitutions were tolerated; although, the authors noted the latter reduced the yield and enantioselectivity of the final product. These indole derivatives are widely present in many nonsteroidal anti-inflammatory drugs such as indomethacin. The indole derivatives synthesized were subjected to virtual screenings for their anti-inflammatory properties and three of them (**213a**, **213b**, and **213c**) showed better results than indomethacin. Mechanistically, this transformation proceeds initially through a Rh-catalyzed ARO via the aromatic amine nucleophile followed by an Pd-catalyzed cyclization.

**Scheme 39:**
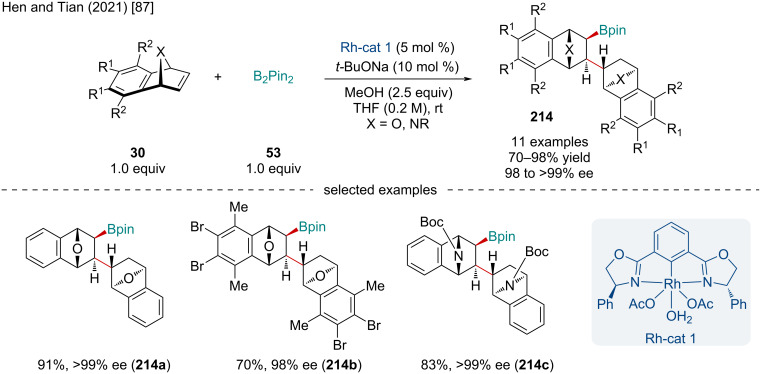
Rh-catalyzed domino carborhodation of heterobicyclic alkenes **30** with B_2_pin_2_ (**53**).

In 2021, He and Tian reported the Rh-catalyzed domino 1,2-carborhodation of heterobicyclic alkenes **30** with B_2_pin_2_ (**53**) (Scheme 39) [87]. EDGs and EWGs were well tolerated on the benzo-fused moiety; however, bridgehead substituents shutdown the reaction. Carbocyclic alkenes, like benzonorbornadiene, failed to produce the desired product leading the authors to conclude the bridging heteroatom of oxa- and azabenzonorbornadiene played a vital role in the carboboration reaction.

**Scheme 40:**
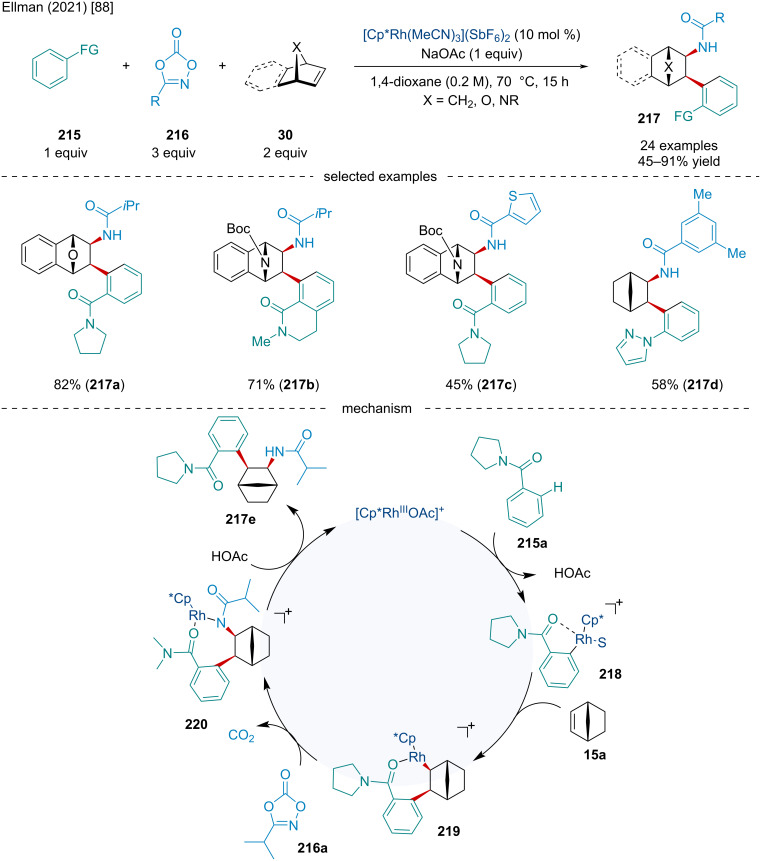
Rh-catalyzed three-component 1,2-carboamidation reaction of bicyclic alkenes **30** with aromatic and heteroaromatic C–H substrates **215** and dioxazolones **216**.

In 2021, Ellman reported a Rh(III)-catalyzed three-component 1,2-carboamidation reaction of bicyclic alkenes **30** with aromatic and heteroaromatic C–H substrates **215** and dioxazolones **216** (Scheme 40) [88]. This reaction was successful with a wide range of directing groups and substituents on the heteroaromatic C–H substrate and a broad range of bicyclic alkenes. Bicyclic diene derivatives like norbornadiene failed to react, likely due to non-productive complexation to the catalyst. Using a chiral cyclopentadiene ligand, the authors showcased an asymmetric variant of the reaction producing 5 enantioenriched products with an average enantiomeric excess of 80% ee. The authors proposed the reaction begins with a concerted metalation–deprotonation of the aromatic C–H substrate **215a** with the Rh(III) catalyst yielding **218**. Migratory insertion of the olefin of **15a** to **218** produces **219**. Subsequently, nitrene insertion of the dioxazolone **216a** to **219** furnishes **220**, which after protodemetalation yields the final product **217e**.

### Palladium-catalyzed reactions

**Scheme 41:**
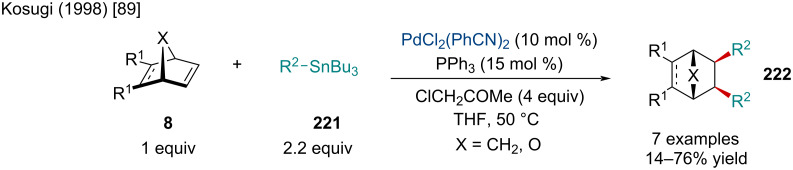
Pd-catalyzed diarylation and dialkenylation reactions of norbornene derivatives.

In 1998, Kosugi and co-workers explored the Pd-catalyzed diarylation and dialkenylation reactions of norbornene derivatives **8** (Scheme 41) [89]. The authors noted the use of chloroacetone was crucial to the reaction as it acted as an exogenous oxidant. Although not perfect, alkenyl stereochemistry was retained for the majority of examples. In the case of (*Z*)-tributylstannylacrylate, the exclusive product was the *exo-cis*-(*E,Z*)-difunctionalized product. Albeit low yielding, heterobicyclic alkenes were tolerated and produced both diarylated and dialkenylated products **222**. On the other hand, benzo-fused heterobicyclic alkenes failed to give the difunctionalized product with the corresponding monofunctionalized ring-opened species being the sole product. Concurrently, the Kang laboratory investigated similar reactivity, disclosing an alternative method for diarylated norbornene derivatives through the three-component coupling of bicyclic alkenes and iodonium salts or diazonium salts with organostannanes, or sodium tetraphenylborate [90]. In 2021, Liu and Chen investigated the use of organoammonium salts and organoboronic compounds as a simple method for the synthesis of diarylated norbornene derivatives [91]. The reaction was also applicable for the addition of benzyl and allyl groups via the organoammonium species.

**Scheme 42:**
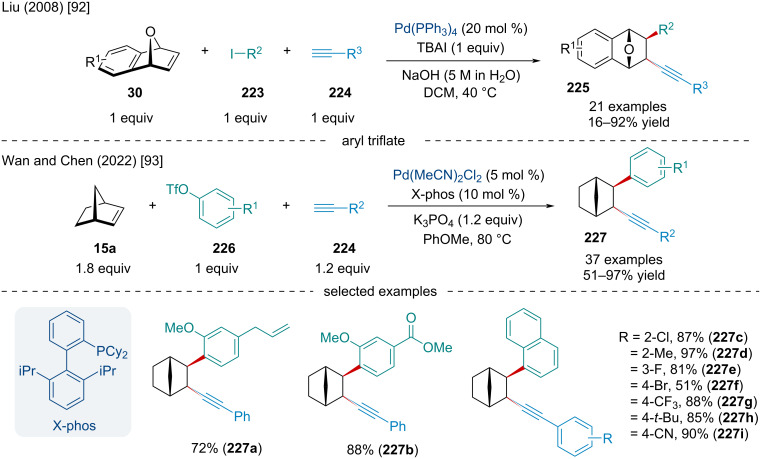
Three-component Pd-catalyzed arylalkynylation reactions of bicyclic alkenes.

In 2008, the Liu laboratory explored the Pd-catalyzed three-component 1,2-arylalkynylation of oxabenzonorbornadiene derivatives **30** (Scheme 42) [92]. Unlike previous reports disclosing the coupling of aryl halides and oxabicyclic alkenes, the authors disclosed the use of 5 M aqueous NaOH to hinder unwanted β-oxygen elimination, promoting difunctionalization of the olefin. The use of the phase-transfer catalyst was paramount, as its removal resulted in little to no conversion. Aryl, alkynyl and alkenyl iodide derivatives, as well as methyl iodide, were shown to operate in the reaction; however, only aryl iodide derivatives routinely gave the desired product in appreciable yield.

In 2022, Wan and Chen explored similar reactivity using aryl triflates (Scheme 42) [93]. The scope of aryl triflates was expansive with derivatives from biologically relevant compounds, like vanillin (**227b**) and eugenol (**227a**), being applicable. Unfortunately, the authors did not expand their scope beyond carbobicyclic frameworks; however, it would be expected the difunctionalization likely does not occur with heterobicyclic alkenes as β-heteroatom elimination could likely be the predominate pathway.

**Scheme 43:**
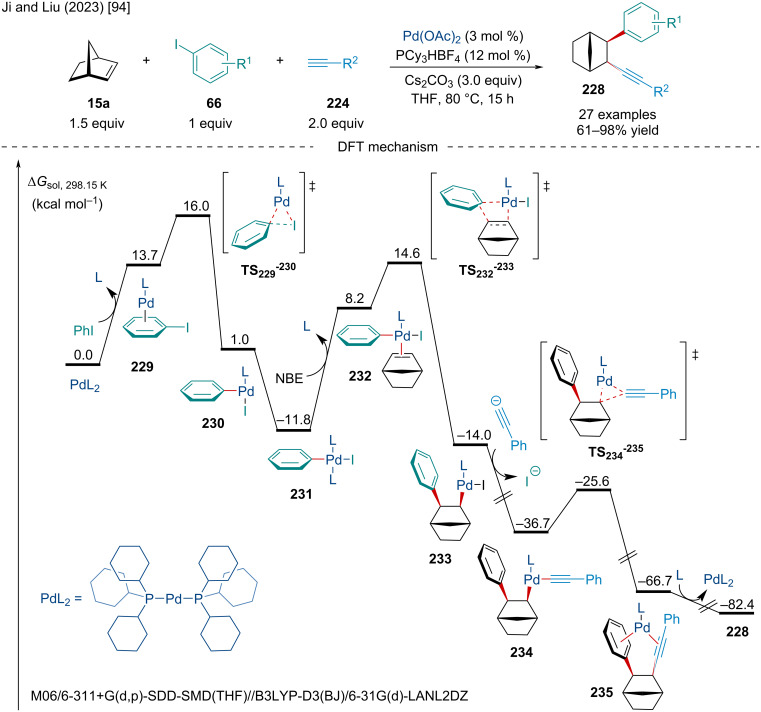
Three-component Pd-catalyzed arylalkynylation reactions of norbornene and DFT mechanistic study.

In 2023, Ji and Liu expanded on the Pd-catalyzed three-component arylalkynylation of oxabenzonorbornadiene derivatives (Scheme 43) [94]. Initially reported by Liu and co-workers in 2006 [95], present conditions were altered to avoid aqueous NaOH, opting for Cs_2_CO_3_. Interestingly, the reaction was applicable to a variety of functional groups, including esters, chlorines, and bromines. In line with similar reports, the scope of bicyclic alkenes was limited with all but a single example being performed on norbornene. DFT calculations were used to explore the reaction mechanism which involves the oxidative addition of the C–I bond, coordination, migratory insertion, transmetalation, and reductive elimination. The authors determined the migratory insertion via **TS_232–233_** is the rate-determining step for the catalytic cycle.

**Scheme 44:**
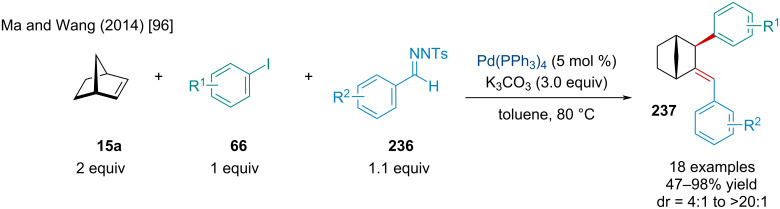
Pd-catalyzed three-component coupling *N*-tosylhydrazones **236**, aryl halides **66**, and norbornene (**15a**).

In 2014, Ma and Wang reported the Pd-catalyzed three-component coupling of *N*-tosylhydrazones, aryl halides, and norbornene (Scheme 44) [96]. The reaction tolerated small substituents on the *N*-tosylhydrazone and aryl halide coupling partners, but the reaction was quite sensitive to *ortho*-substitution and steric bulk. Generally, the reaction gave the corresponding product in good yield and excellent diastereoselectivity; however, a few substrates produced diastereomeric ratios of 3:1. As the propensity for an *exo*-selective migratory insertion is well understood, it is surprising some products displayed such poor selectivity. As such, this may indicate some form of stereoisomerization rather than a poorly selective migratory insertion. In the following year, Xu and Liang reported a reaction involving the same three coupling partners [97]. By altering the reaction conditions, the authors observed the first palladium-catalyzed ring opening of norbornene to prepare methylenecyclopentane derivatives via an unusual β-carbon elimination.

**Scheme 45:**
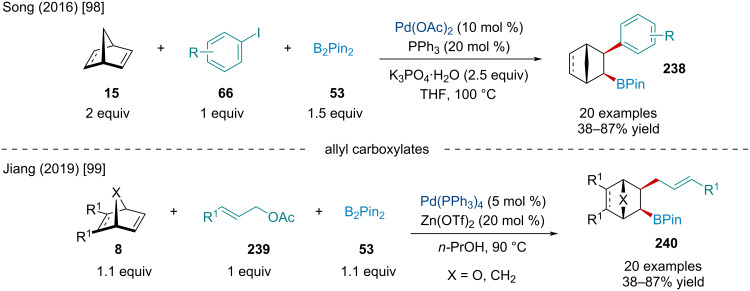
Pd-catalyzed arylboration and allylboration of bicyclic alkenes.

In 2016, the Song laboratory reported the Pd-catalyzed arylboration of norbornene derivatives (Scheme 45) [98]. Generally, electron-rich aryl halides afforded the product in a higher yield than those bearing electron-withdrawing groups. Moreover, the reaction was amenable to heteroaromatic iodides, but yields were diminished. The authors showed aryl bromides were tolerated albeit with slightly diminished yields relative to their iodide-containing counterparts. The scope of bicyclic alkenes was mainly limited to norbornene with a single example using norbornadiene.

In 2019, Jiang and co-workers expanded on this chemistry and revealed allyl carboxylates can be used as the nucleophilic partner in carboborylation difunctionalization reactions (Scheme 45) [99]. Besides allyl acetates, the authors revealed formates, propionates, and butanoates were able to afford the desired product; however, allyl bromides and chlorides failed. Unfortunately, the reaction was sensitive to the bicyclic alkene used; norbornadiene and 2,3-diester-substituted norbornene were unable to undergo the transformation. Surprisingly, oxabenzonorbornadiene was amenable and afforded the difunctionalized product in 44% yield rather than a ring-opened product.

**Scheme 46:**
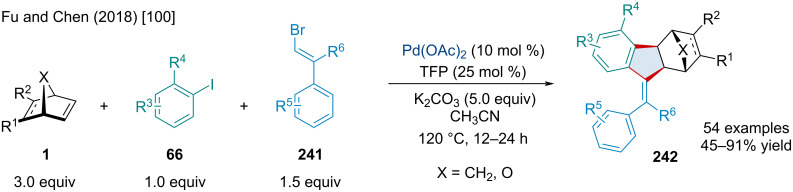
Pd-catalyzed, three-component annulation of aryl iodides **66**, alkenyl bromides **241**, and bicyclic alkenes **1**.

In 2018, Fu and Chen reported the Pd-catalyzed, three-component annulation of aryl iodides **66**, alkenyl bromides **241**, and bicyclic alkenes **1** (Scheme 46) [100]. Similar reports by the Lautens [101] and Perumal [102] laboratories have demonstrated the use of norbornene derivatives for the synthesis of tetrasubstituted olefins; however, limited work has been done for the synthesis of trisubstituted olefins. The authors noted *ortho*-substituted iodobenzenes delivered products in a greater yield compared to their strictly *meta-* or *para*-substituted counterparts like due to the elimination of complex byproducts. Typically, reactions gave products with very high *Z* stereoselectivity. The authors demonstrated the methodology could be applied towards the synthesis of tetrasubstituted olefins as well, giving the desired product in moderate to good yields. This methodology avoided the use of highly substituted internal alkynes, a substrate which can be more difficult to synthesis than its alkenyl bromide counterpart. The reaction is applicable to other bicyclic alkenes although with slightly diminished yields compared to norbornene. Unsymmetrically substituted bicyclic alkenes bearing relatively sensitive functionalities, such as -CHO and -CN, worked, albeit with no regioselectivity.

**Scheme 47:**
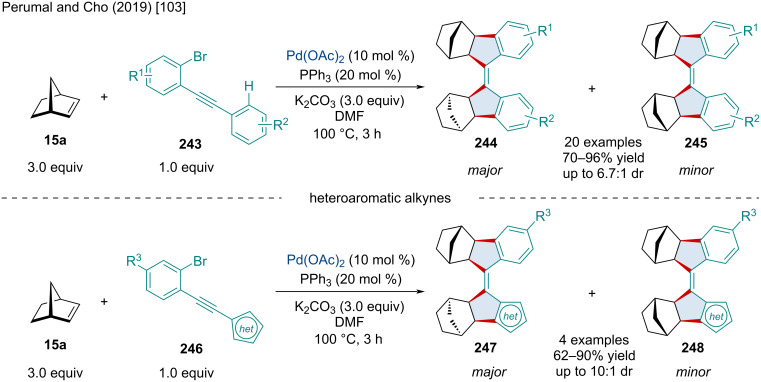
Pd-catalyzed double insertion/annulation reaction for synthesizing tetrasubstituted olefins.

In 2019, Perumal and Cho reported a Pd-catalyzed double insertion/annulation reaction for synthesizing tetrasubstituted olefins (Scheme 47) [103]. Mechanistically, the transformation involves the formation of four new C–C bonds through three consecutive carbopalladations and a C–H activation. Unlike the anticipated *exo* migratory insertion seen almost exclusively in these types of systems, the authors noted the second norbornene moiety undergoes an *endo*-selective reaction, as confirmed through X-ray crystallography. The diastereoselectivity of the reaction was limited due to the production of the unanticipated *exo-endo* adduct **244**/**247** which was formed as the major product while the thermodynamically more stable *exo*-*exo* product **244**/**248** was only ever achieved in minor yields. The methodology was explored with a broad scope of aryl substituents revealing the robustness of the reaction. Additionally, heteroaromatic alkynes **246** were found to be tolerable but gave slightly diminished yields.

**Scheme 48:**
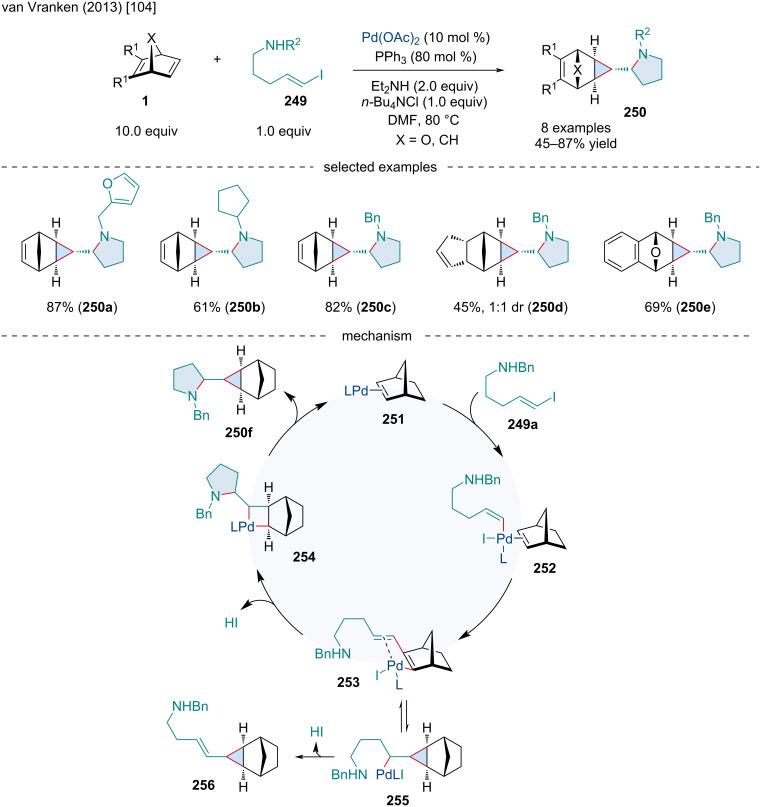
Pd-catalyzed aminocyclopropanation of bicyclic alkenes **1** with 5-iodopent-4-enylamine derivatives **249**.

In 2013, van Vranken and co-workers reported the Pd-catalyzed aminocyclopropanation of bicyclic alkenes **1** with 5-iodopent-4-enylamine derivatives **249** (Scheme 48) [104]. The reaction was effective for a range of *N*-substituted derivatives **249**; however, the reaction was sensitive to steric bulk. With large groups, like *N*-adamantyl, only modest yields of the desired pyrrolidine product were obtained, owing to the formation of the vinylcyclopropane side product. Other bicyclic alkenes were amenable, including an example with an oxabicyclic alkene which underwent the desired reaction rather than the anticipated β-oxygen elimination side reaction. The mechanism for this transformation involves the oxidative addition of the alkenyl iodide to the Pd(0) and side-on coordination to the olefin **252**, followed by the migratory insertion of the bicyclic alkene to afford complex **253**. Aminopalladation of the olefin affords **254** which undergoes a reductive elimination to generate the final product **250**. In the case of the vinylcyclopropane side product, complex **253** preferentially undergoes a carbopalladation to generate a cyclopropane intermediate **255** which undergoes a β-hydride elimination to give **256**.

**Scheme 49:**
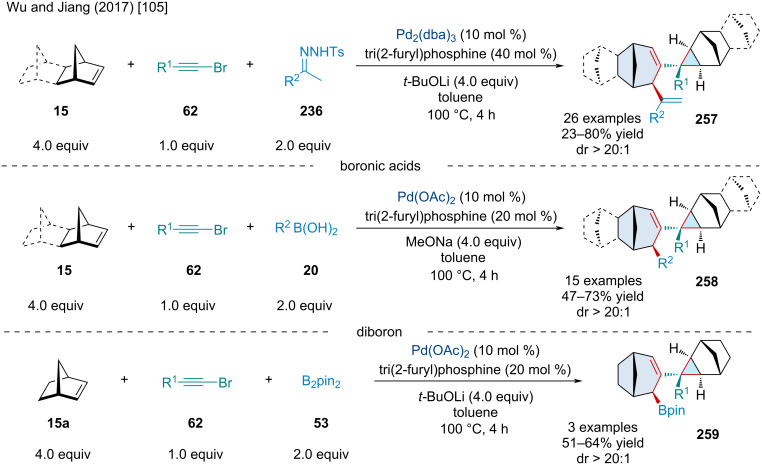
Pd-catalyzed, three-component coupling of alkynyl bromides **62** and norbornene derivatives **15** with electrophilic trapping agents.

In 2017, Wu and Jiang reported a Pd-catalyzed, three-component coupling of alkynyl bromides **62**, norbornene derivatives **15**, with electrophilic trapping agents (Scheme 49) [105]. Mechanistically, the transformation begins with the oxidative addition of the alkynyl bromide to the Pd(0) catalyst. From here, four consecutive carbopalladation reactions ultimately end up producing an alkylpalladium intermediate which undergoes a β-carbon elimination to afford a Pd–π-allyl species. First, the authors captured this π-allyl species with *N*-tosylhydrazone derivatives **236**. Notably, alkynyl bromides **62** bearing electron-withdrawing groups typically afforded the desired product in greater yield. The scope of the *N*-tosylhydrazones **236** was expansive with electronic substituents having little effect on the reaction. Heteroaromatic *N*-tosylhydrazones **236** were applicable but gave diminished yields. Moving on, the authors showed the Pd–π-allyl species can be trapped with boronic acids **20**. Like the *N*-tosylhydrazones **236**, the substituents on the boronic acid had little effect on the reaction. Lastly, the authors demonstrated the use of B_2_pin_2_ **53** to capture the Pd–π-allyl species.

**Scheme 50:**
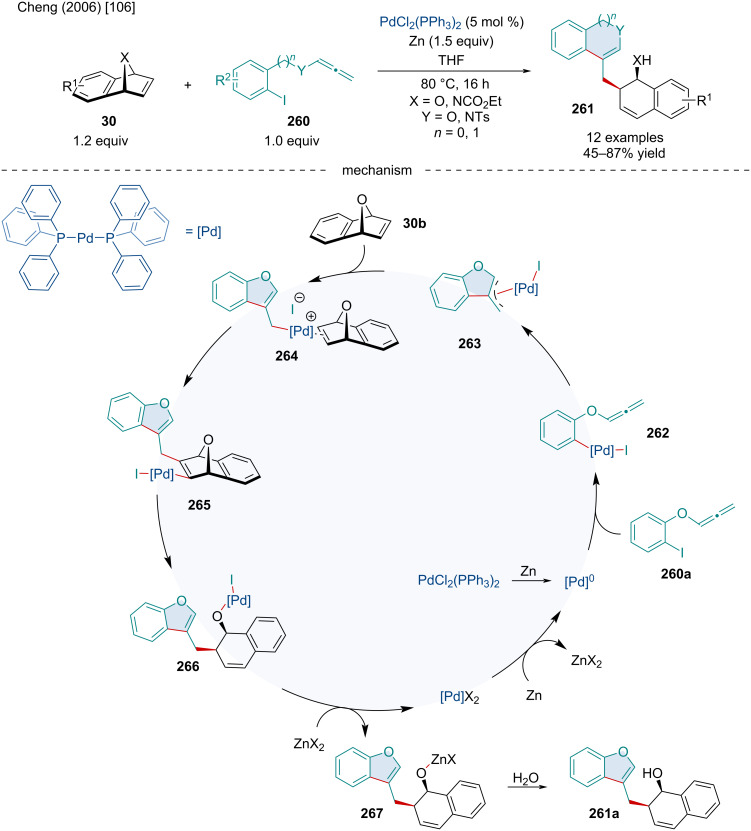
Pd-catalyzed intramolecular cyclization/ring-opening reaction of heterobicyclic alkenes **30** with 2-iodophenoxyallenes **260**.

In 2006, the Cheng group investigated the Pd-catalyzed intramolecular cyclization/ring-opening reaction of heterobicyclic alkenes **30** with 2-iodophenoxyallenes **260** (Scheme 50) [106]. Surprisingly, the efficacy of the reaction was more susceptible to derivatization of the benzo-fused moiety with sterically demanding functionalities rather than altering the electronics, as seen with severely diminished yields with phenanthrene-fused oxabicyclic alkenes. The reaction was unaffected by the identity of the bridging heteroatom with both oxa- and aza-bridging atoms performing equally as well; although, the latter was only explored a single time. Altering the tether length of the allene moiety seemed to mildly affect the reaction with 5-membered rings being formed in slightly greater yields compared to their 6-membered counterparts. Mechanistically, this reaction operates similarly to other cyclization/capture chemistry seminally presented by Griggs [107–108]. First, the Pd(II) catalyst is reduced to the Pd(0) active catalyst with Zn metal. Oxidative addition of the aryl iodide **260a** to Pd(0) gives **262** which can side-on coordinate with the allenyl group. Intramolecular migratory insertion affords the Pd–π-allyl species **263** which can side-on coordinate to the *exo* face of the bicyclic alkene **264**. Rather than dissociation of the iodide ligand to generate a cationic Pd center, it has also been proposed the loss of a phosphine ligand could allow for the generation of a free coordination site. Migratory insertion affords intermediate **265** which undergoes a β-oxygen elimination to **266**. Transmetalation with ZnCl_2_ affords the zinc alkoxide **267** which is hydrolyzed to give the final product **261a**. Alternatively, Zn metal could reduce Pd(II) intermediate **266** to Pd(0) directly, bypassing the transmetalation step.

**Scheme 51:**
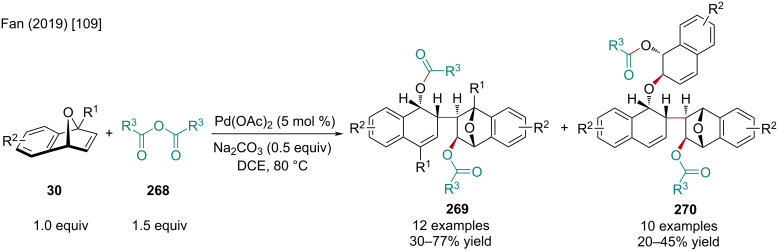
Pd-catalyzed dimer- and trimerization of oxabenzonorbornadiene derivatives **30** with anhydrides **268**.

In 2019, the Fan group explored the Pd-catalyzed dimer- and trimerization of oxabenzonorbornadiene derivatives **30** with anhydrides **268** (Scheme 51) [109]. The authors noted electron-deficient oxabenzonorbornadiene derivatives resulted in diminished product yields. When electron-rich bicyclic alkenes were used, the trimer **270** to dimer **269** ratio was increased. When applied to unsymmetrically substituted bicyclic alkenes, the authors propose the dimerized product was formed as a single regioisomer, as evaluated by ^1^H NMR, with no trimerization observed.

**Scheme 52:**
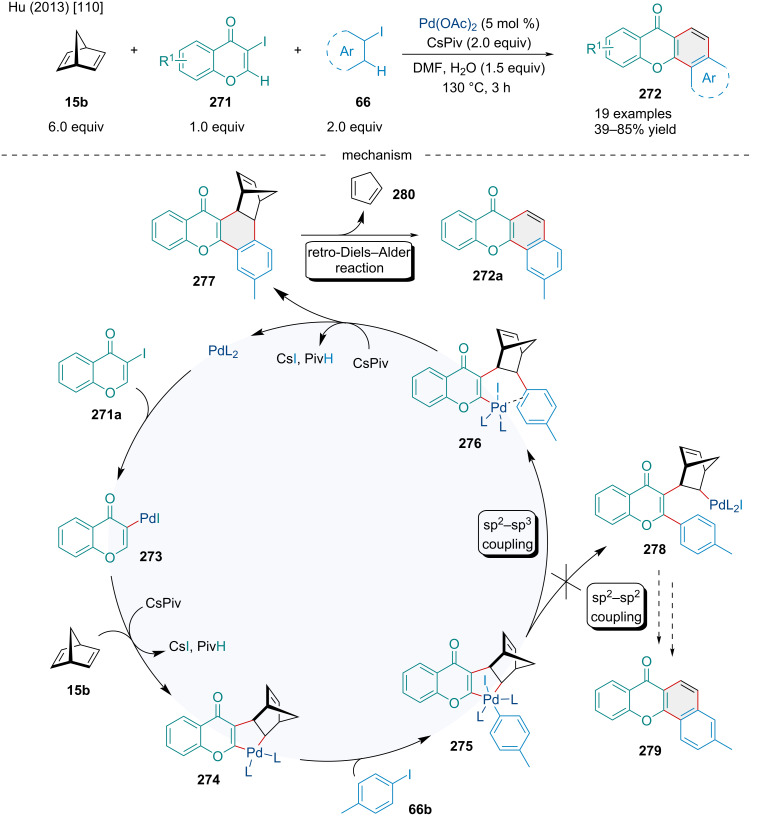
Pd-catalyzed Catellani-type annulation and retro-Diels–Alder of norbornadiene **15b** yielding fused xanthone derivatives **272**.

In 2013, the Hu laboratory developed a method to form annulated xanthones **272** using norbornadiene (**15b**), 3-iodochromones **271**, and aryl iodides **66** via a Catellani-type reaction (Scheme 52) [110]. The authors proposed a mechanism beginning with the oxidative addition of Pd(0) to **271a**, followed by migratory insertion across norbornadiene (**15b**) and alkenyl C–H activation of the chromone ring, furnishing the palladacycle **274**. The oxidative addition of **274** to the aryl iodide **66b** yields a Pd(IV) species **275** that can undergo reductive elimination by either an sp^2^–sp^3^ or sp^2^–sp^2^ coupling event. The authors probed the regioselectivity of this step using *p*-iodotoluene and, based on the product, concluded that only sp^2^–sp^3^ coupling occurred. The resulting intermediate **276** undergoes an aryl C–H activation step and a subsequent reductive elimination yielding a norbornadiene-fused xanthone derivative **277**, which forms the final product **272a** via a retro-Diels–Alder reaction. The reaction was generally tolerant of substituted 3-iodochromones; however, substituted aryl iodides were shown to have significant effects. Electron-donating *para*-substituents and bulky *ortho*-substituents resulted in lower yields while bulky *meta*-substituents could be used to influence the regioselectivity of the C–H activation step. The scope was limited to dienes because of the necessity for a retro-Diels–Alder to furnish the desired product but a norbornane-fused xanthone was also produced in 82% yield.

**Scheme 53:**
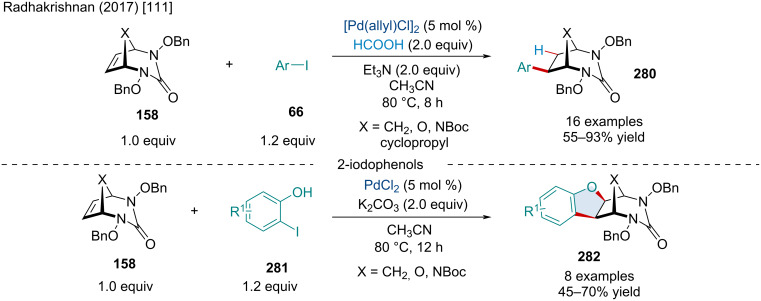
Pd-catalyzed hydroarylation and heteroannulation of urea-derived bicyclic alkenes **158** and aryl iodides **66**.

In 2017, Vijayan et al. investigated both the Pd-catalyzed hydroarylation and annulation of urea-derived bicyclic alkenes **158** using aryl iodides **66** (Scheme 53) [111]. In both reactions, the 1,2-migratory insertion of palladium across the olefin resulted in a palladacycle intermediate that was further reacted, either via hydride-donor or an *ortho*-directing group, to form the hydroarylated **280** or annulated products **282**, respectively. For this reason, the formic acid additive was necessary in the hydroarylation but was left out in the annulation to promote capture by the phenolic directing group. The hydroarylation gave moderate to good yields with EWGs and EDGs alike, as well as accommodating *ortho*-substituents. It was also tolerant of spiro-, furan-derived, and *N*-protected pyrrole-derived bicyclic alkenes, all giving similar yields. The heterobicyclic alkenes were shown to be compatible with the annulation as well, though they resulted in slightly reduced yields compared to the carbocyclic examples. Although the authors focused on the use of an alcohol directing group for the annulation to furnish dihydrobenzofurans, they also provided a simple example using methyl bromide and nitrile directing groups giving indane and indanone products in similar yields.

**Scheme 54:**
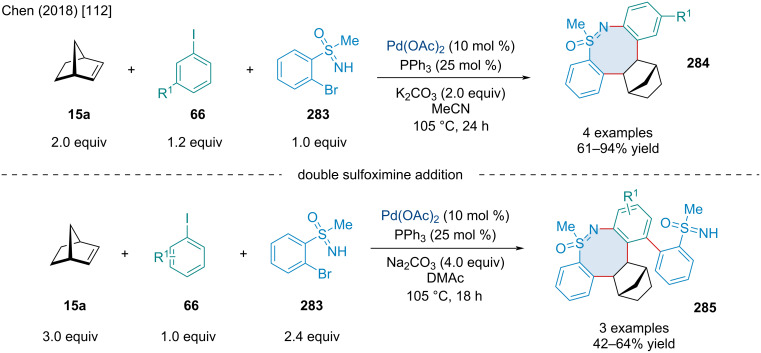
Access to fused 8-membered sulfoximine heterocycles **284**/**285** via Pd-catalyzed Catellani annulation cascades.

In 2018, the Chen laboratory explored a Pd/norbornene cocatalyzed Catellani annulation reaction of phenyl iodides **66** and NH-sulfoximines **283** in an attempt to produce dibenzothiazines [112]. Though they were successful in this effort, they also reported accessing eight-membered sulfoximine heterocycles when norbornene was not extruded, which was accomplished in two distinct ways (Scheme 54) [112]. The first requires aryl iodides with *meta*-EWGs, which was shown by DFT calculation to favor sp^2^–sp^3^ coupling over sp^2^–sp^2^ coupling. This coupling step prevents the extrusion of norbornane later without restricting the Pd catalyst’s access to the sulfoximine directing group, thus allowing the formation of the 8-membered heterocyclic product **284**. The other method requires slightly modified conditions, mainly by increasing the equivalents of NH-sulfoximines **283**, and for the phenyl iodides have two *ortho*-hydrogens. The second hydrogen allows for sequential C–H activation after the standard sp^2^–sp^2^ coupling, again preventing the extrusion of norbornene, and creating a Pd(II) species that undergoes oxidative addition with the extra sulfoximines provided, eventually forming a heterocycle bearing two sulfoximine moieties **285**. Understandably, the presented examples are limited, as these products were of secondary interest to the authors but yields of up to 94% for product **284** and from 42% to 64% for product **285** were reported.

**Scheme 55:**
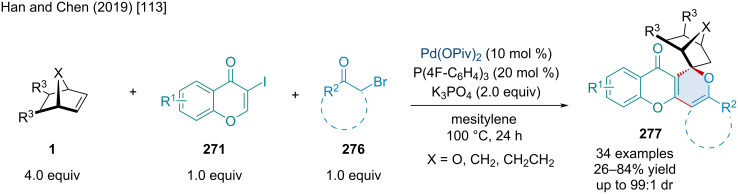
Pd-catalyzed 2,2-bifunctionalization of bicyclic alkenes **1** generating spirobicyclic xanthone derivatives **277**.

Six years after the work of Hu and co-workers producing annulated xanthones, Yang et al. completed a 2,2-bifunctionalization of bicyclic alkenes **1** to produce xanthone derivatives bearing spirobicyclic moieties **277** (Scheme 55) [113]. This was achieved via a Pd-catalyzed [2 + 3 + 1] annulation of 3-iodochromones **271**, bromoacetones **276**, and bicyclic alkenes **1**. The reaction generally afforded good yield and diastereoselectivity even across the wide swathe of functionalized substrates and few bicyclic alkenes tested and provided a good yield (71%) at the gram scale.

**Scheme 56:**
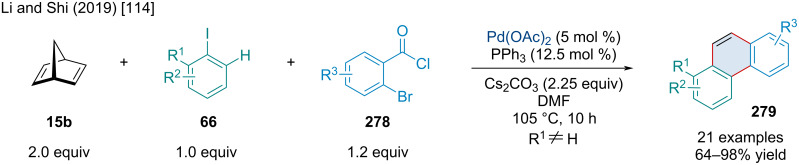
Pd-catalyzed Catellani-type annulation and retro-Diels–Alder of norbornadiene (**15b**) producing substituted phenanthrenes **279**.

In 2019, Zhong et al. reported a method to produce phenanthrene derivatives **279** using 2-iodotoluenes **66**, *o*-bromobenzoyl chlorides **278**, and norbornadiene (**15b**). This method proceeds through a Pd-catalyzed Catellani reaction; however, norbornadiene extrusion is avoided via the loss of the acyl chloride group as CO, allowing the formation of a 7-membered palladacycle, reductive elimination, and subsequent retro-Diels–Alder reaction to the phenanthrene **279** (Scheme 56) [114]. This was an improvement over past methods that used less reactive *ortho*-haloaryl carboxylic acids which required harsher conditions and longer reaction times to optimally perform. The reaction was shown to be tolerant of diverse functionality, providing excellent yields barring a couple notable examples; 1-iodonaphthalene (64%) and *o*-iodonitrobenzene (75%). The authors were also able to demonstrate its efficacy at the gram scale with a yield of 88%.

**Scheme 57:**
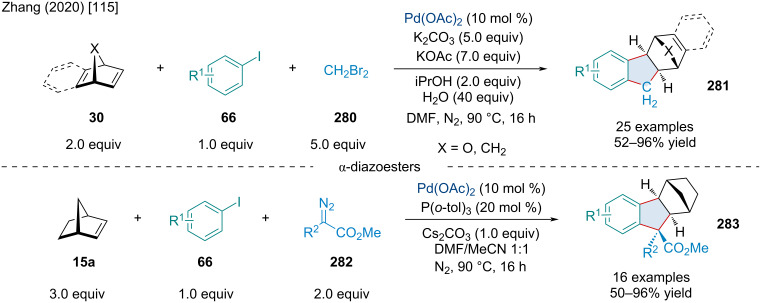
Pd-catalyzed [2 + 2 + 1] annulation furnishing bicyclic-fused indanes **281** and **283**.

In 2020, Zhang and colleagues explored a three-component Pd-catalyzed annulation reaction furnishing norbornane-fused indanes **281** (Scheme 57) [115]. This reaction sees an aryl iodide **66** coupled to a bicyclic alkene **30** to produce a 5-membered palladacycle intermediate that is then captured by the third reagent, either methylene bromide (**280**) or an α-diazoester **282**. A reduced yield was seen in the absence of iPrOH, so it was kept as an additive with the authors proposing it functions as a reductant, reducing Pd(II) to the active catalyst Pd(0). A great variety of examples using methylene bromide (**280**) were reported, including using a few different bicyclic alkenes **30**, with up to 96% yield. A similar variety of examples with similar yields were shown using α-diazoesters **282**, however, only norbornene proved suitable in this case with heterobicyclic alkenes unable to afford the desired product.

**Scheme 58:**
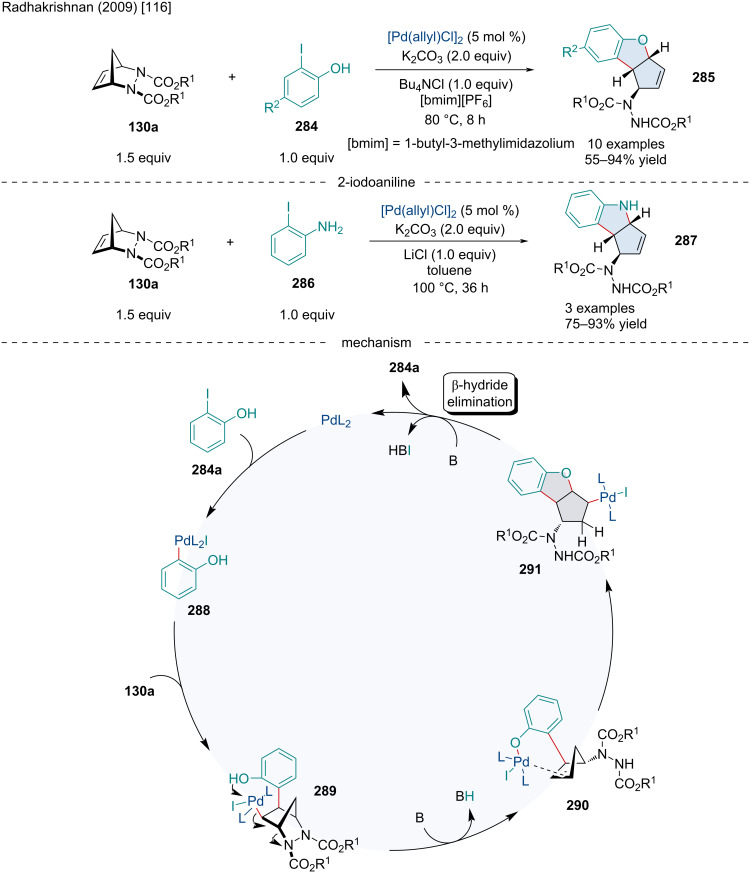
Pd-catalyzed ring-opening/ring-closing cascade of diazabicyclic alkenes **130a**.

In 2009, the Radhakrishnan laboratory investigated a Pd-catalyzed annulation of diazabicyclic alkenes **130a**, and 2-iodophenols **284** or 2-iodoaniline (**286**) towards fused benzofuran **285** or indole **287** products (Scheme 58) [116]. The reaction begins with the oxidative addition of Pd(0) into the aryl iodide **284a**, followed by migratory insertion across the bicyclic alkene to form **289**. Base-assisted addition of the alcohol and β-nitrogen elimination forms a ring-opened cyclopentene intermediate **290** which then undergoes oxypalladation and β-hydride elimination, furnishing the benzofuran product **284a**. The authors noted that in the absence of the Bu_4_NCl additive the reaction did not work. The authors hypothesized the chloride ions are important for regenerating and stabilizing the Pd(0) species. While only a handful of examples were reported, it was demonstrated that diazabicyclic alkenes with bulkier ester groups caused reduced yields.

**Scheme 59:**
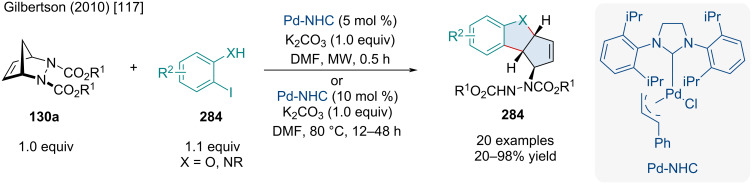
Pd-NHC-catalyzed cyclopentannulation of diazabicyclic alkenes **130a**.

One year later, the Gilbertson laboratory expanded on this annulation reaction, increasing its efficiency and significantly decreasing the reaction time using tweaked conditions and microwave irradiation (Scheme 59) [117]. They also significantly increased the scope of the reaction, providing many examples with up to 98% yield, and utilizing N-substituted anilines to create N-substituted indoles **284**. The authors were also able to apply their methodology to an acetal-protected vanillin derivative, producing the corresponding benzofuran with 90% yield.

**Scheme 60:**
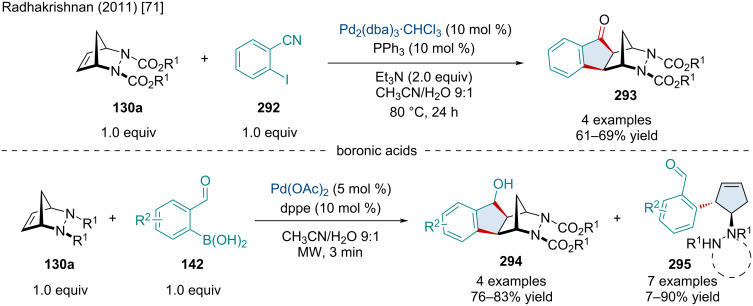
Pd-catalyzed annulation cascade generating diazabicyclic-fused indanones **292** and indanols **294**.

Two years after their previous work, the Radhakrishnan group explored a non-ring-opening annulation utilizing 2-iodobenzonitrile (**292**) and 2-formylphenylboronic acids **142** to access diazabicyclic-fused indanones **293** and indanols **294** (Scheme 60) [71]. The authors noted the addition of base increased the yield of 2-iodobenzonitrile (**292**) reactions but reduced it for those with 2-formylphenylboronic acids **142**. Only a few examples producing indanones **293** were presented showing very small changes in yield with different diazabicyclic esters. Different N-substituted triazolinedione-derived bicyclic alkenes were also tested but failed, likely due to their base sensitivity. The annulation reaction yielding indanols **294** was seen to produce the 3,4-disubstituted cyclopentene **295** in ratios of about 1:9 when the diazabicyclic alkenes **130a** were used. However, when using the N-substituted triazolinedione-derived bicyclic alkenes the 3,4-disubstituted cyclopentene **294** could be produced exclusively in yields of up to 90%.

**Scheme 61:**
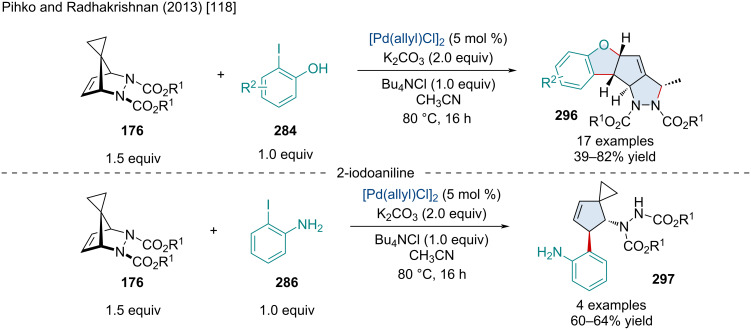
Pd-catalyzed skeletal rearrangement of spirotricyclic alkenes **176** towards large polycyclic benzofuran derivatives **296**.

In 2013, Pihko and Radhakrishnan revisited their 2009 annulation reaction using 2-iodophenols **284** and 2-iodoaniline (**286**) in an attempt to access larger polycyclic compounds **296** through the use of spirotricyclic olefins **176** (Scheme 61) [118]. It is proposed that the reaction follows a similar ring-opening/ring-closing mechanism to their 2009 report (Scheme 58), but the cyclopropane moiety allows a second ring opening and the subsequent generation of a π-allyl–palladium complex. This complex undergoes an intramolecular nucleophilic attack by hydrazine, forming the fourth fused ring. When the methodology was applied to 2-iodoaniline (**286**), the anticipated polycyclic product was not formed; instead, *trans*-disubstituted spiro[2,4]hept-4-enes **297** were formed. A variety of substituted 2-iodophenols **284** were tested showing significantly reduced yields with *para*-EWGs, emphasizing the importance of an electron-rich alcohol directing group.

**Scheme 62:**
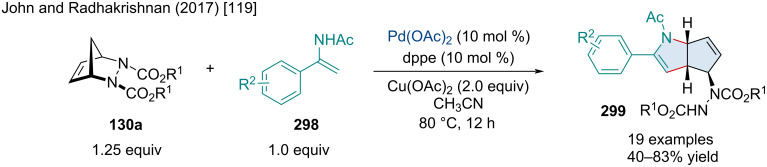
Pd-catalyzed oxidative annulation of aromatic enamides **298** and diazabicyclic alkenes **130a**.

In 2017, the Radhakrishnan group investigated another ring-opening/ring-closing reaction of diazabicyclic alkenes **130a**, synthesizing cyclopenta[*b*]pyrroline derivatives **299** using aromatic enamides **298** (Scheme 62) [119]. Since the reaction begins with an alkenyl C–H activation, forming a 6-membered palladacycle intermediate with amide oxygen chelation, Cu(OAc)_2_ was added as an oxidant to regenerate Pd(II). Afterwards, the transformation progresses similarly to their 2009 report (Scheme 58). The 6-membered palladacycle will undergo migratory insertion into the diazabicyclic alkene **130a** which after a β-nitrogen elimination, adds to the amide via the nitrogen atom. Aminopalladation forms the C–N bond that produces the fused pyrroline moiety in the product **299**. The authors reported several examples using substituted aromatic enamides finding that EWGs were well tolerated while EDGs significantly reduced yields. When applied at the gram scale, the desired product was produced with 60% yield.

**Scheme 63:**
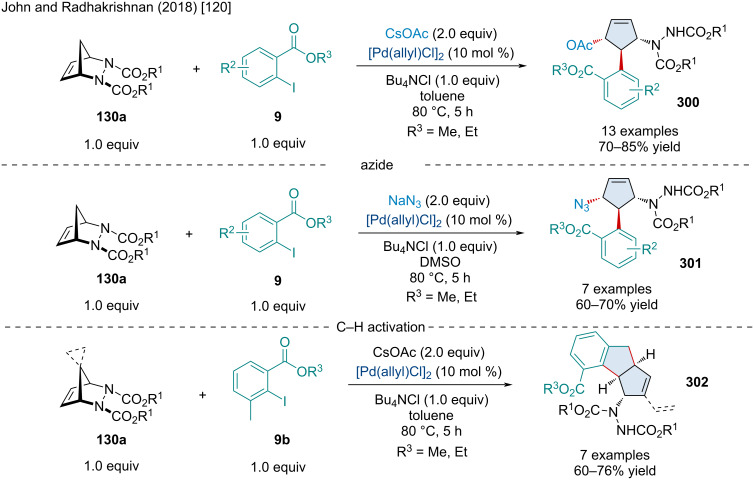
Accessing 3,4,5-trisubstituted cyclopentenes **300**, **301**, **302** via the Pd-catalyzed domino reaction of diazabicyclic alkenes **130a** and 2-iodobenzoates **9**.

In 2018, Radhakrishnan and colleagues again expanded on their past work, attempting to produce 3,4,5-trisubstituted cyclopentenes **300** from diazabicyclic alkenes **130a** and 2-iodobenzoates **9** (Scheme 63) [120]. The authors proposed a charged fused-oxane intermediate is produced after the ring-opening/ring-closing sequence, as anticipated in 2009 (Scheme 58), whose eventual breakdown furnishes a π-allyl–palladium complex which undergoes nucleophilic attack by the acetate or azide anion. Several examples were reported, ranging a 60–85% yield, showing minimal electronic influence by 2-iodobenzoate substituents. However, another reaction path was observed when 2-iodo-3-methylbenzoate (**9a**) was used, producing a cyclopentene-fused indane **302**. The authors suggested that the mechanism of this reaction follows the same steps until the formation of the π-allyl–palladium complex, which can undergo cyclopalladation via benzylic C–H activation of the 3-methyl group, and subsequently reductive elimination to yield the fused indane product **302**.

**Scheme 64:**
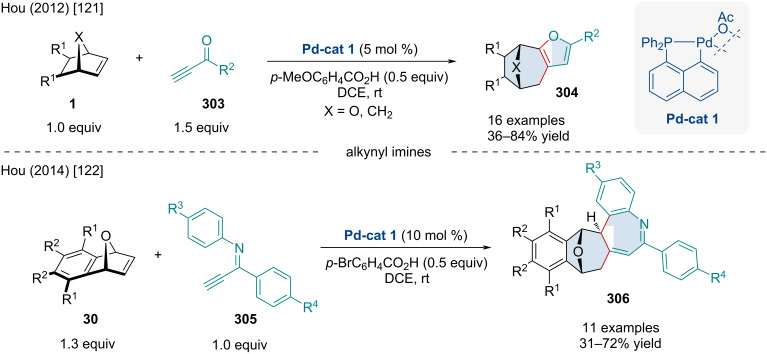
Palladacycle-catalyzed ring-expansion/cyclization domino reactions of terminal alkynes and bicyclic alkenes.

In 2012, Ge et al. investigated a palladacycle-catalyzed reaction furnishing highly substituted fused furans **304** using bicyclic alkenes **1** and terminal ynones **302** (Scheme 64) [121]. The authors noted the reaction was sensitive to the identity of both basic and acidic additives, as bases tended to slow down reactions while stronger acids typically produced only a trace amount of the desired product **304**. Eventually, the authors discovered their goldilocks additive, settling on *p*-methoxybenzoic acid which showed a significant increase in yield. The reaction was generally tolerant of a variety of substituted terminal ynones **303** and bicyclic alkenes **1**, as well as norbornene (**15a**) and norbornadiene (**15b**). Two years later, this methodology was expanded by the same group, using terminal alkynyl imines **305** to access polycyclic 5*H*-benzo[*b*]azepines **306** (Scheme 64) [122]. The authors reported low yields when R^2^ or R^3^ were weak EWGs and no reaction with strong EWGs at R^3^, somewhat restricting the scope of the reaction.

**Scheme 65:**
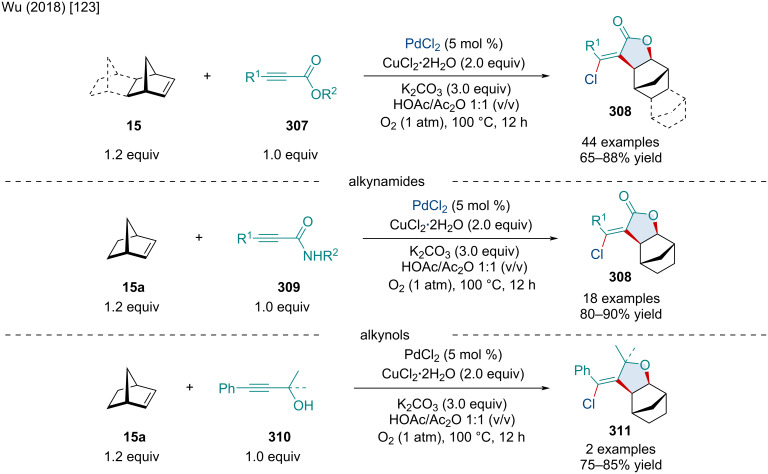
Pd-catalyzed carboesterification of norbornene (**15a**) with alkynes, furnishing α-methylene γ-lactones **308** and tetrahydrofurans **311**.

In 2018, the Jiang laboratory explored a Pd-catalyzed carboesterification reaction, using bicyclic alkenes **15** and alkynoates **307**, ynamides **309**, and alkynols **310** to produce α-methylene γ-lactone **308** and tetrahydrofuran derivatives **311** (Scheme 65) [123]. The reaction was shown to be functionally tolerant, boasting a large number of high yielding examples. Largely, the authors noted substitution of the ester or the amide moiety had little influence on the reaction. Only two examples were reported for the reaction of the alkynol **310**, albeit in good yields.

## Conclusion

Over the last two decades, there has been remarkable progress in transition-metal-catalyzed domino reactions of homo- and heterobicyclic alkenes. Bicyclic alkenes can be exploited in two ways. Firstly, through the release of ring-strain energy which drives the reaction forward under milder conditions compared to strainless alkene counterparts. Secondly, the stereochemically well-defined, dual-faced nature of these systems can be exploited to synthesize highly stereoselective products.

Multicomponent domino reactions can be challenging due to selectivity issues, but recent advancements have provided straightforward protocols for the construction of complex molecules with multiple carbon–carbon and carbon–heteroatom bonds in a single step. When participating in a well-orchestrated domino sequence, these bicyclic alkenes can quickly generate highly functionalized products with extreme stereo-, regio-, and enantioselectivity.

Currently, a majority of transition-metal-catalyzed domino reactions use simple carbocyclic alkenes, such as norbornene, as the propagative π-system of choice, limiting its relevance. To see further advancements in this field, it is necessary to expand the scope to include more heterobicyclic alkenes and understand their fundamental reactivity. As heterobicyclic alkenes have the tendency to undergo some form a β-heteroatom elimination which can prematurely terminate a cascade, their use requires more thought. However, altering coupling partners, reaction conditions, and the metal center have all been used to promote difunctionalization of heterobicyclic alkenes while hindering β-heteroatom elimination. We hope this comprehensive overview of bicyclic alkene chemistry will drive further advancements in the area of transition-metal-catalyzed domino reactions.
